# Evidence for extensive hybridisation and past introgression events in feather grasses using genome-wide SNP genotyping

**DOI:** 10.1186/s12870-021-03287-w

**Published:** 2021-11-01

**Authors:** Evgenii Baiakhmetov, Daria Ryzhakova, Polina D. Gudkova, Marcin Nobis

**Affiliations:** 1 Institute of Botany, Faculty of Biology, Jagiellonian University, Gronostajowa 3, 30-387 Kraków, Poland; 2 Research laboratory ‘Herbarium’, National Research Tomsk State University, Lenin 36 Ave., 634050 Tomsk, Russia; 3 Department of Biology, Altai State University, Lenin 61 Ave., 656049 Barnaul, Russia

**Keywords:** Feather grasses, Hybridisation, Introgression, Integrative taxonomy, Genome-wide genotyping, DArTseq, Divergence-time estimation, Population structure

## Abstract

**Background:**

The proper identification of feather grasses in nature is often limited due to phenotypic variability and high morphological similarity between many species. Among plausible factors influencing this issue are hybridisation and introgression recently detected in the genus. Nonetheless, to date, only a bounded set of taxa have been investigated using integrative taxonomy combining morphological and molecular data. Here, we report the first large-scale study on five feather grass species across several hybrid zones in Russia and Central Asia. In total, 302 specimens were sampled in the field and classified based on the current descriptions of these taxa. They were then genotyped with high density genome-wide markers and measured based on a set of morphological characters to delimitate species and assess levels of hybridisation and introgression. Moreover, we tested species for past introgression and estimated divergence times between them.

**Results:**

Our findings demonstrated that 250 specimens represent five distinct species: *S. baicalensis*, *S. capillata*, *S. glareosa*, *S. grandis* and *S. krylovii*. The remaining 52 individuals provided evidence for extensive hybridisation between *S. capillata* and *S. baicalensis*, *S. capillata* and *S. krylovii*, *S. baicalensis* and *S. krylovii*, as well as to a lesser extent between *S. grandis* and *S. krylovii*, *S. grandis* and *S. baicalensis*. We detected past reticulation events between *S. baicalensis*, *S. krylovii*, *S. grandis* and inferred that diversification within species *S. capillata*, *S. baicalensis*, *S. krylovii* and *S. grandis* started ca. 130–96 kya. In addition, the assessment of genetic population structure revealed signs of contemporary gene flow between populations across species from the section *Leiostipa*, despite significant geographical distances between some of them. Lastly, we concluded that only 5 out of 52 hybrid taxa were properly identified solely based on morphology.

**Conclusions:**

Our results support the hypothesis that hybridisation is an important mechanism driving evolution in *Stipa*. As an outcome, this phenomenon complicates identification of hybrid taxa in the field using morphological characters alone. Thus, integrative taxonomy seems to be the only reliable way to properly resolve the phylogenetic issue of *Stipa*. Moreover, we believe that feather grasses may be a suitable genus to study hybridisation and introgression events in nature.

**Supplementary Information:**

The online version contains supplementary material available at 10.1186/s12870-021-03287-w.

## Background

The proper delimitation of species plays an important role in taxonomy as well as in studies related to speciation, biogeography and ecology, leading to effective conservation and management of biodiversity. In the last two decades, traditional approaches relying mostly on morphological features have been supplemented by molecular data that boosted the discovery of new species. Although one estimate of the number of plant species is around 298,000 [1], recently it has been shown that the plant kingdom is comprised of at least 374,000 taxa [2]. Nowadays, many systematicists emphasise the need to apply multidisciplinary data, so-called integrative approaches or integrative taxonomy [3]. For instance, information from a variety of disciplines, e.g., morphology, biochemistry, cytogenetics and ‘omics studies, increases the reliability and validity in identifying taxa [4–6].

To date, among the molecular methods, DNA barcoding has been a widely utilised tool to identify taxa at different levels aiming not only to facilitate revisionary taxonomy, but also to broaden our understanding of molecular phylogenetics and population-level variations [7–9]. Among standard plant markers, chloroplast regions *rbcL* and *matK* and the nuclear internal transcribed spacer (ITS) locus have been proposed for DNA barcoding of land plants [10, 11]. Additionally, several non-coding plastid regions have been suggested as supplementary loci where further resolution is required [10].

*Stipa* is one of the largest genera in the grass subfamily Pooideae (Poaceae), currently comprising nearly 150 cool-season species with C_3_ photosynthesis common in Eurasia and North Africa [12, 13]. Based on ITS and the plastid *trnK* region, the genus has been proven to be monophyletic [14, 15]. Nonetheless, the traditional barcodes are not able to validate the sectional subdivision in *Stipa* proposed, e.g., by Tzvelev [16, 17] or Freitag [18]. Recently, the nuclear intergenic spacer (IGS) region [19] and marker sets derived from whole chloroplast genomes of 19 species [20] were proposed for phylogenetic studies of feather grasses. Although these markers are more phylogenetically informative in comparison to the previously used barcodes, they are still unable to discriminate all taxa, causing unresolved nodes in the reconstructed trees [19, 20].

One of the plausible explanations for this unresolved branching in *Stipa* is that many feather grasses are of hybrid origin [16, 21–23]. Presently, hybridisation is considered to be widespread among at least 25% of plant taxa, mostly the youngest species [24]. This phenomenon is often accompanied by introgression via repeated backcrossing to one or both parental species that can lead to diversification and speciation [25, 26]. In grasses, hybrid speciation has been explicitly studied at the genome level, e.g., in *Triticum* [27] and *Brachypodium* [28]. Nonetheless, previously many hybrids and introgressed individuals were characterised exclusively based on morphology that limited their successful identification in nature [29, 30]. In addition, hybridisation may lead new organisms not only to intermediate traits of parental species but also to extreme, or transgressive, phenotypes [31] that complicate their proper taxonomic treatment. In feather grasses, the hypothesis of hybrid origin of some species was initially tested using multivariate morphological analyses [23, 32] and, more recently, applying molecular markers among genetically closely related species in the *Stipa heptapotamica* complex [33] as well as within two genetically distant species, *S. krylovii* and *S. bungeana* [34]. Furthermore, due to the usage of integrative taxonomic approaches, it was shown that some *Stipa* taxa, previously assigned to *S. richteriana* and *S. grandis*, appeared to be cryptic species [33, 35]. Thus, taking into account that ca. 30% of feather grasses could be of hybrid origin [13] and that cryptic species are present, integrative taxonomy seems to be the only reliable way to properly resolve the phylogenetic issue of *Stipa*.

Importantly, the advent of next generation sequencing technologies, primarily Illumina, and the continuously reducing sequencing cost have facilitated the implementation of genomic data in studies of non-model organisms. For instance, high-throughput techniques based on restriction enzymes, e.g., RADseq [36] and genotyping-by-sequencing [37], which have been foremost used in agricultural species [38], are currently widely applied in phylogenetics and studies related to hybridisation in many wild plant genera with little or no previous genomic information [39–41]. Recently, a promising result has also been demonstrated in *Stipa* where the usage of the DArTseq technique resulted in an increased number of markers that was several 100-fold higher than in the previous genomic studies [34].

**Fig. 1:**
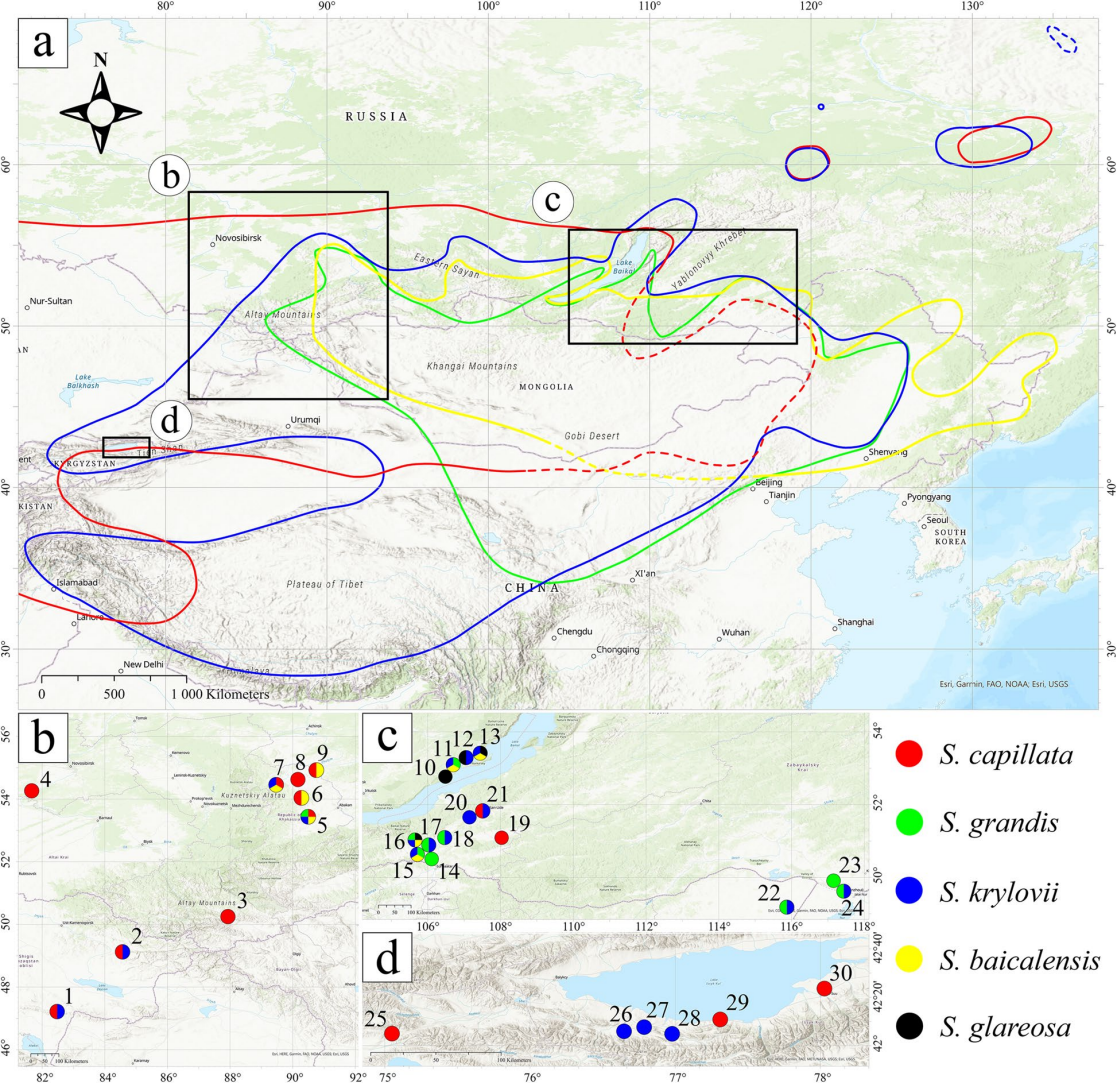
The general distribution map of (**a**) *S. baicalensis* (yellow), *S. capillata* (red), *S. grandis* (green), *S. krylovii* (blue) and sampling locations (**b**) in East Kazakhstan and southwestern Siberia (Russia), (**c**) in southeastern Siberia and (**d**) in Eastern Kyrgyzstan. The dashed lines indicate hypothetical borders. The coloured circles depict species found in the numbered locations. The exact coordinates of the locations are presented in the Supplementary Table S1

During field studies on steppe communities, we observed high morphological variability in populations of genetically related plants. In particular, we noticed that some specimens of feather grasses representing *S. capillata*, *S. grandis* and *S. krylovii* seemingly share mixed morphological characteristics between these species, while taxon *S. baicalensis* is frequently observed within populations of the aforesaid taxa and resembles an intermediate phenotype between *S. grandis* and *S. krylovii* or *S. capillata* and *S. krylovii*. The above-mentioned species have wide distribution ranges (Fig. 1 and Supplementary Table S1). Specifically, *S. capillata* is the most widespread taxon within the genus, grows on the dry grasslands and is common in Siberia, Western Asia and is also present in a limited number of refugia in Europe and North Africa. Two species, *S. baicalensis* and *S. grandis*, share similar ranges in southwestern Siberia, in the Baikal region, in the south part of Zabaykalsky Krai, in Mongolia and in northeastern China; *S. baicalensis* is also present in the south part of the Russian Far East, whereas *S. grandis* occurs in Central China and Tibet. Finally, *S. krylovii* grows in Siberia, Mongolia, China, Northern Nepal, Southern Tajikistan, Eastern Kazakhstan and Eastern Kyrgyzstan [13, 42].

We hypothesise that the observed variability in *S. baicalensis*, *S. capillata*, *S. grandis* and *S. krylovii* is due to the presence of interspecific hybrids and that may lead to species misidentification based on the current descriptions of these taxa [16, 43–45]. Thus, in the current study, we aim to use an integrative taxonomy approach to (1) delimitate species and test if *S. baicalensis* is a hybrid between *S. grandis* × *S. krylovii* or *S. capillata* × *S. krylovii*; (2) assess levels of hybridisation and introgression (if present) between the examined taxa and populations at the molecular level; (3) estimate divergence times between the studied taxa; (4) obtain insight into the extent of hybridisation between these species at the morphological level and (5) assess whether morphological characters can be used to identify hybrids in the field.

## Results

### DNA-based species delimitation

**Fig. 2:**
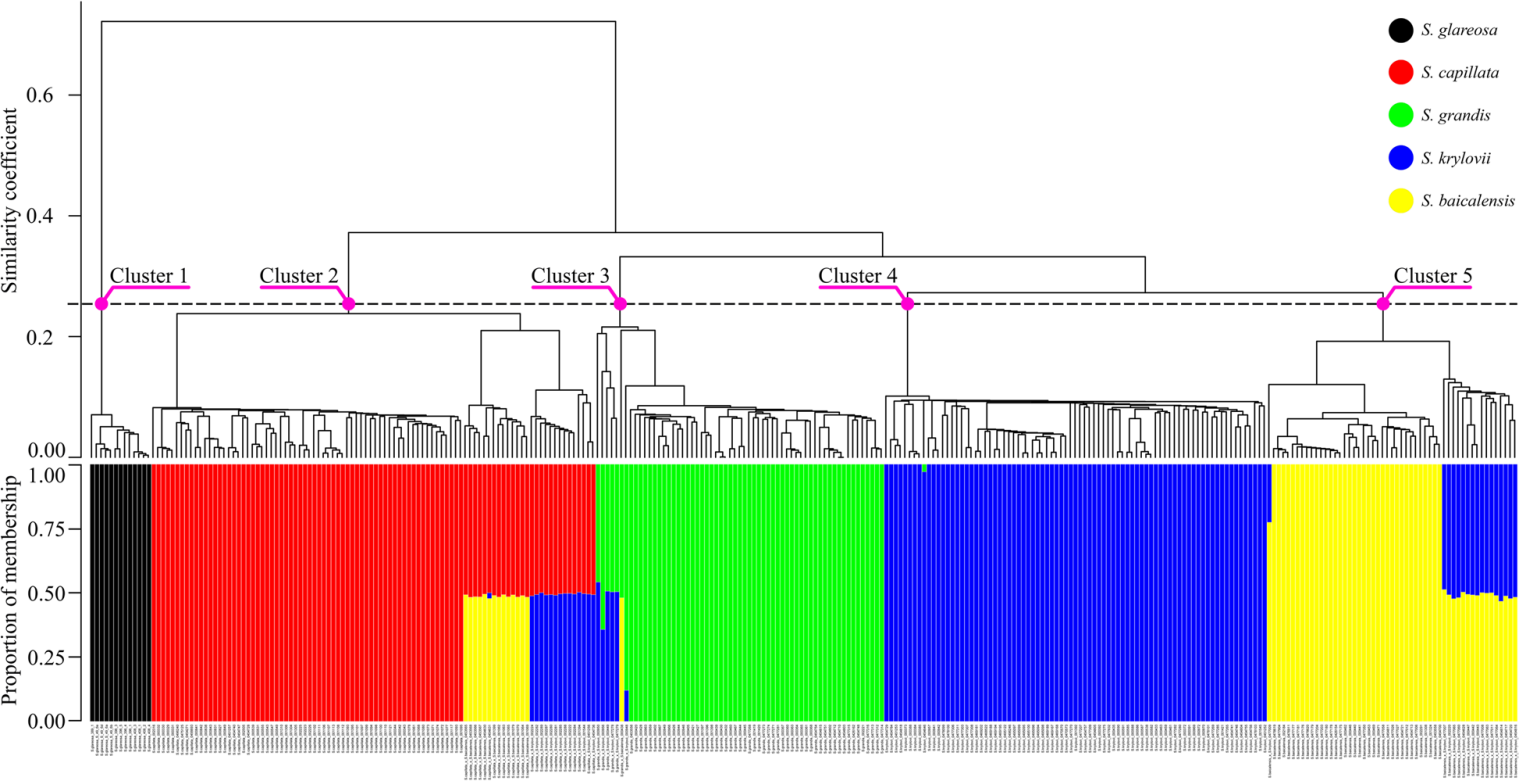
The UPGMA dendrogram (at the top) aligned with the best supported fastSTRUCTURE model K = 5 (on the bottom). The genetic distance was calculated using the Jaccard Similarity Coefficient (y-axis, top). Individuals are represented by coloured bars according to the proportion of membership (y-axis, bottom) of a genotype to the respective cluster

The DArTseq technique was applied to obtain a total of 8660 SNP markers to infer the genetic structure of 302 *Stipa* specimens. Firstly, analyses of genetic clustering with an unweighted pair group method using arithmetic average (UPGMA) and fastSTRUCTURE revealed five major clades corresponding to morphospecies *S. glareosa*, *S. capillata*, *S. grandis*, *S. krylovii* and *S. baicalensis* (Fig. 2). According to the fastSTRUCTURE analysis, the first and the fourth clades consisted exclusively of pure specimens of *S. glareosa* and *S. krylovii*, respectively. The remaining clades beside pure specimens of *S. capillata*, *S. grandis* and *S. baicalensis* included hybrid individuals. In particular, the second clade comprised pure specimens of *S. capillata* and the admixed individuals *S. capillata × S. baicalensis* and *S. capillata × S. krylovii.* The third cluster consisted of pure specimens of *S. grandis* and hybrids *S. grandis × S. krylovii* and *S. grandis × S. baicalensis*. The fifth clade included pure specimens of *S. baicalensis* and the admixed individuals *S. baicalensis × S. krylovii*.

In total, fastSTRUCTURE inferred 52 individuals with an admixture of two genetic clusters including an exception of *S. capillata × S. baicalensis* (0454631) that had a minor proportion (0.02) of a third cluster representing *S. krylovii*. Among pure individuals only one specimen of *S. krylovii* (0454646) had an insignificant admixed proportion (0.03) of *S. grandis*. Noteworthy, the vast majority of admixed individuals (49 or 94%) had a proportion of membership in the range from 0.46 to 0.54 indicating F1 hybrids or later generations of hybrids that have no backcrossing to the parental species. The remaining admixed samples represented: (1) one individual (0477009) was formed by a 0.78–0.22 admixture between *S. baicalensis* and *S. krylovii* evidencing a first-generation backcross (F1*× S. baicalensis*), (2) one individual (000948) was shared between *S. grandis* (0.88) and *S. krylovii* (0.12) indicating a second-generation backcross (first-generation backcross × *S. grandis*), (3) one individual (000956) was admixed between *S. grandis* (0.64) and *S. krylovii* (0.36) that may suggest a first-generation backcross (F1*× S. grandis*) or a more complex backcross to *S. grandis* via different intermediate combinations.

**Fig. 3:**
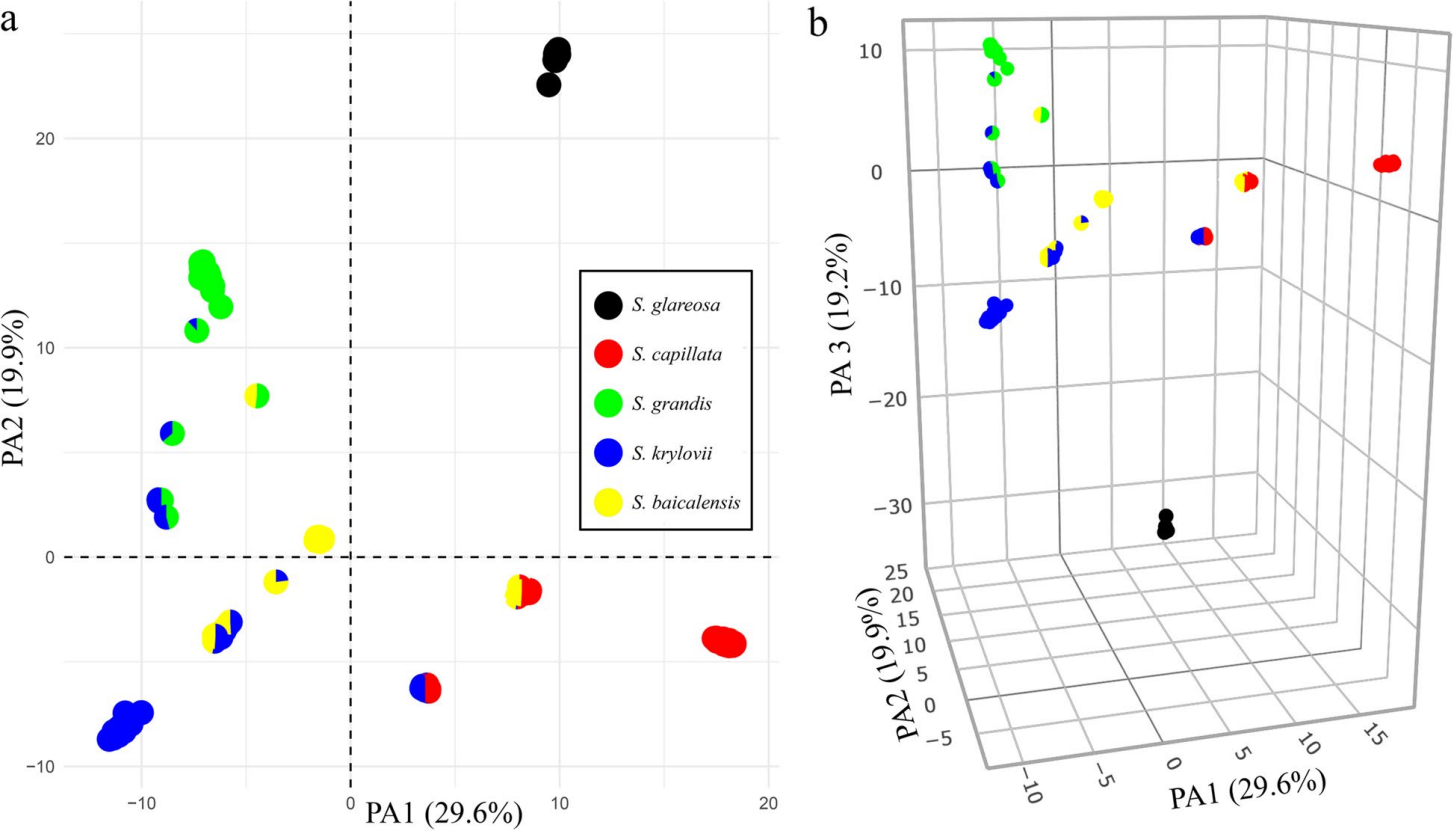
The PCoA plot based on genetic distances between samples. **a** The plot of the two principal axes. **b** The plot of the three principal axes. The pie charts represent the proportions of membership established by fastSTRUCTURE for the best K = 5

A consistent result was also found with a principal coordinates analysis (PCoA). The first three axes explained 29.6, 19.9 and 19.2% of the total genetic divergence within the studied taxa, respectively. According to the PCoA, pure individuals were grouped into five markedly differentiated groups correspondingly to their taxonomic classifications (Fig. 3; an interactive version of the three-dimensional plot can be accessed via https://plot.ly/~eugenebayahmetov/40/). The remaining hybrids F1 had intermediate positions between their parental species. Two hybrid individuals *S. grandis* × *S. krylovii* (000948 and 000956) were grouped closer to *S. grandis* reflecting a higher proportion of membership with the first taxon established earlier with fastSTRUCTURE. Similarly, an admixed individual *S. baicalensis* × *S. krylovii* (0477009) with the proportion of 0.78 and 0.22 was closer to *S. baicalensis*.

### Hybrid generation identification

**Fig. 4:**
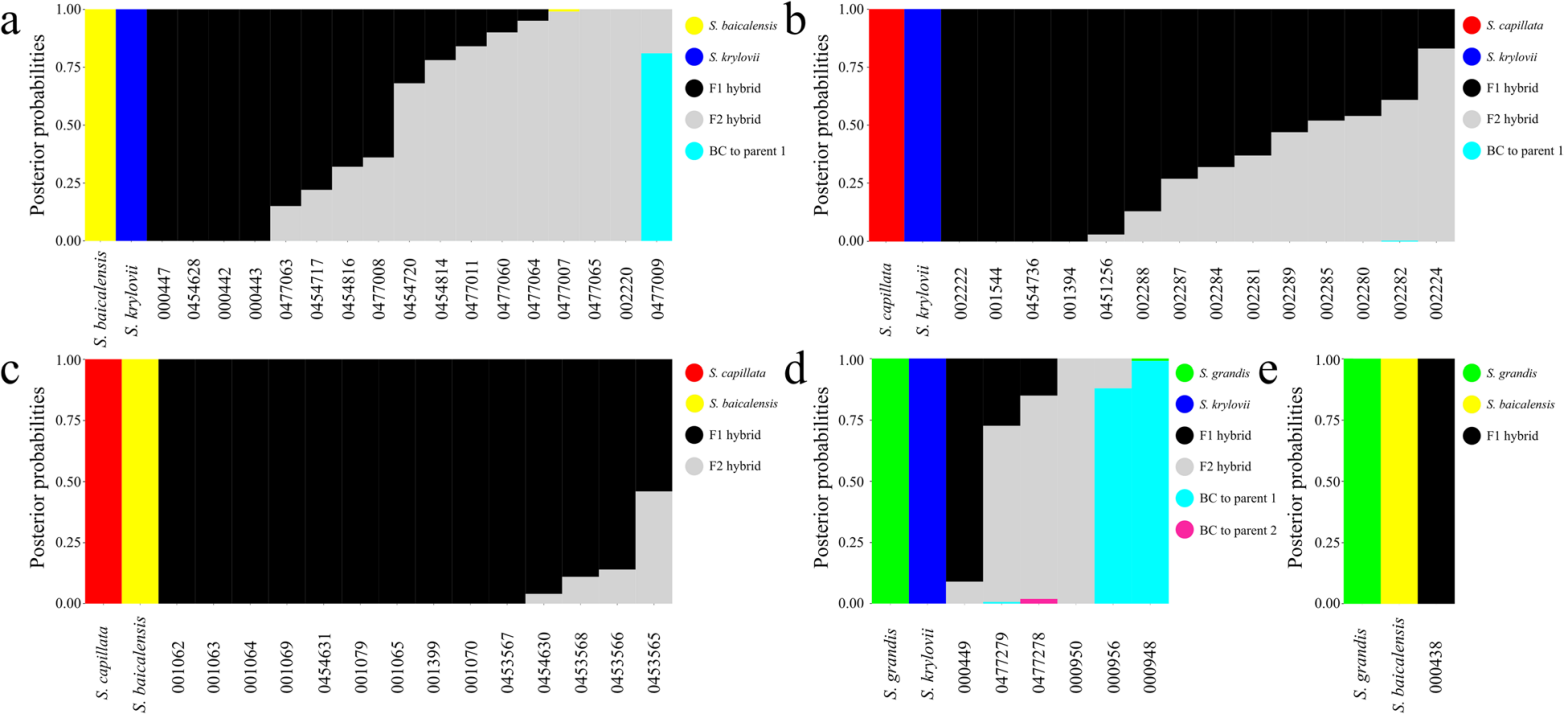
The assignment of *Stipa* taxa into four hybrid classes according to the posterior probabilities (y-axis) inferred in NewHybrids. **a** *S. baicalensis* × *S. krylovii*, (**b**) *S. capillata* × *S. krylovii*, (**c**) *S. capillata* × *S. baicalensis*, (**d**) *S. grandis* × *S. krylovii*, (**e**) *S. grandis* × *S. baicalensis*. Hybrid classes are coloured by black (F1 hybrid), grey (F2), cyan (backcross to the first parental species, BC to parent 1) and pink (backcross to the second parental species, BC to parent 2)

The NewHybrids analysis revealed a more complex pattern of hybridisation than it was inferred with fastSTRUCTURE. Among 16 admixed specimens of *S. baicalensis* × *S. krylovii*, previously assigned as F1, six individuals with posterior probabilities (PP) in a range of 0.84 and 1.00 were identified being F2 (F1× F1) hybrids suggesting that F1 hybrids are able to reproduce further. One specimen, *S. baicalensis* × *S. krylovii* (0477009), was proven to be a first-generation backcross (F1*× S. baicalensis*) having PP of 0.81. In addition, five mixed individuals had PP between two categories (F1 and F2 hybrids) in a range of 0.22–0.78 suggesting uncertainty in the assignment (Fig. 4a and Supplementary Table S2). These mixed assignment individuals may represent a more advanced hybrid generation than can be detected by NewHybrids. Within 14 hybrids of *S. capillata* × *S. krylovii*, previously assigned to F1 hybrids, we detected six individuals of F1 (PP of 0.87–1.00) and one specimen was identified as an F2 hybrid with PP of 0.83. The remaining seven individuals had mixed assignments in a range of 0.39–0.73 for the F1 class and 0.27–0.61 for the F2 class, respectively (Fig. 4b). The analysis also demonstrated that 13 out 14 hybrids of *S. capillata* × *S. baicalensis* were F1 (PP of 0.86–1.00) and one individual remained unclassified sharing PP (0.54 and 0.46) between F1 and F2 categories (Fig. 4c). Among *S. grandis* × *S. krylovii* only one F1 hybrid was detected (PP of 0.91), two individuals were assigned to the F2 class (PP of 0.83 and 1.00) and two specimens were classified as first generation backcrosses (F1× *S. grandis*) having PP of 0.88 and 0.99. Although the specimen 000948 was inferred to be an F1 backcross, it is more plausible that it represents rather a second-generation backcross established by fastSTRUCTURE due to NewHybrids being unable to detect more advanced backcrosses than F1. Additionally, one individual had mixed assignments between the F1 (PP of 0.27) and F2 (PP of 0.72) classes (Fig. 4d). Finally, the only hybrid detected for *S. grandis* × *S. baicalensis* appeared to be a first generation hybrid with PP of 1.00 (Fig. 4e).

### Testing for introgression

A total of 6894 SNP markers were used to test for reticulation events between the studied taxa. Due to five different admixed combinations detected by fastSTRUCTURE and NewHybrids, we tested all possible four-species combinations regardless of their phylogenetic positions (Fig. 2). The results of the *f*_4_ statistic suggest no gene flow between *S. capillata* and the remaining species because of negligible deviations from the expected 50/50 ratio of BABA/ABBA patterns and the lowest Z-scores of any tests (Table 1). This finding disagrees with the presence of contemporary hybrids *S. capillata* × *S. krylovii* and *S. capillata* × *S. baicalensis* inferred with fastSTRUCTURE and NewHybrids. Nonetheless, it can be explained by the fact that all identified admixed individuals were excluded from this analysis. On the other hand, introgression events were suggested between *S. grandis* and *S. baicalensis* (combinations 5 and 11), *S. grandis* and *S. krylovii* (combinations 4, 6, 7 and 8), *S. krylovii* and *S. baicalensis* (combinations 9 and 12). Additionally, when *S. grandis*, *S. krylovii* and *S. baicalensis* were analysed together (combinations 4, 7 and 10) the ratio of BABA/ABBA patterns were either almost equal (combination 10) or relatively lower (combinations 4 and 7) compared to the other tests that indicated gene flow among these species. One potential explanation is that these species are involved in introgression at the same rate, which theoretically cancel out each other.

**Table 1:** Test for introgression between the studied species using 6894 SNPs

No	B	C	D	nBABA	nABBA	*f*_4_	*Z*-score
1	*S. capillata*	*S. grandis*	*S. krylovii*	11	11	−0.000094	−0.187
2	*S. capillata*	*S. grandis*	*S. baicalensis*	15	17	−0.000284	−0.494
3	*S. capillata*	*S. krylovii*	*S. baicalensis*	13	15	−0.000190	−0.293
4	*S. grandis*	*S. krylovii*	*S. baicalensis*	23	64	−0.006000	−4.570
5	*S. grandis*	*S. capillata*	*S. baicalensis*	130	17	0.016400	10.000
6	*S. grandis*	*S. krylovii*	*S. capillata*	11	166	−0.022400	−9.090
7	*S. krylovii*	*S. baicalensis*	*S. grandis*	64	30	0.004870	3.530
8	*S. krylovii*	*S. capillata*	*S. grandis*	166	11	0.022500	8.910
9	*S. krylovii*	*S. baicalensis*	*S. capillata*	15	136	−0.017600	−10.200
10	*S. baicalensis*	*S. krylovii*	*S. grandis*	23	30	−0.001130	−1.260
11	*S. baicalensis*	*S. capillata*	*S. grandis*	130	15	0.016700	10.400
12	*S. baicalensis*	*S. krylovii*	*S. capillata*	13	136	−0.017800	−10.800

Outgroup (A) for all tests was *S. glareosa*; nBABA, number of BABA patterns; nABBA, number of ABBA patterns. Standard error in all tests was < 0.01. Negative *f*_4_ and *Z*-score < −3 indicate gene flow between B and C, positive *f*_4_ and *Z*-score > 3 suggest reticulation events between B and D.

### Population differentiation

**Fig. 5:**
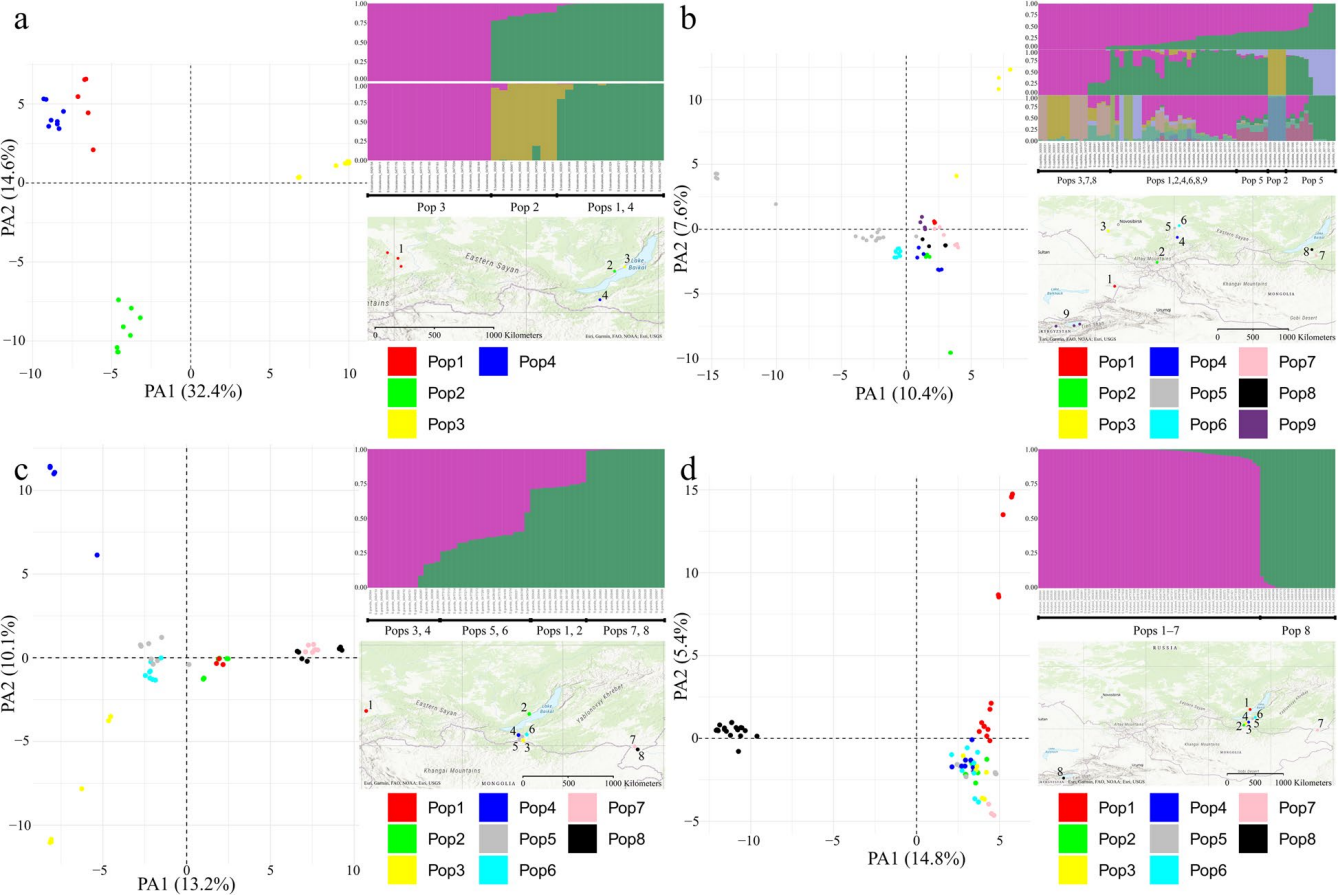
PCoA plots, best supported STRUCTURE models and localities of the studied populations across four species. **a** *S. baicalensis*. **b** *S. capillata.* **c** *S. grandis.* **d** *S. krylovii*

A total of 3483 SNP markers were used to investigate the genetic differentiation in populations of *S. baicalensis*, 6288 SNPs in *S. capillata*, 4635 SNPs in *S. grandis* and 6912 SNPs in *S. krylovii* (Supplementary Fig. S1). The pairwise *F*st values demonstrated strong differentiation among four populations of *S. baicalensis*, while the results of STRUCTURE and PCoA revealed two and three genetic clusters, respectively (Fig. 5a and Supplementary Fig. S2), where populations 1 and 4 are merged (*F*st of 0.32, Supplementary Table S3) regardless of the fact that the distance between them is more than 1000 km. Additionally, the second most likely K according to STRUCTURE was K = 3 indicating that this number of clusters is also a likely option. Relatively strong differentiation was also shown for populations of *S. capillata* with an exception of populations 5 and 6 with a moderate *F*st value of 0.13, suggesting potential gene flow. According to the PCoA, almost all individuals were grouped together excluding population 3 and a few specimens of populations 2 and 5. Nonetheless, the first two axes of PCoA explained only 18% of the total genetic divergence within the specimens. On the other hand, STRUCTURE supported K = 4 as the best fitting model, while two and nine clusters were also among the probable options (Fig. 5b and Supplementary Fig. S2). Within *S. grandis*, evidence for weak differentiation was shown for geographically close populations 5 and 6 (*F*st of 0.10) as well as for 7 and 8 (*F*st of 0.08), while the first two axes of the PCoA explained 23.3% of the variation and revealed the close genetic relationship between geographically distant populations 1 and 2 (*F*st of 0.42). In addition, based on the second axis of the PCoA, populations 3 and 4 were distant to each other as well as to the remaining populations (Fig. 5c). Further, the STRUCTURE analysis suggested that K = 2 was the most probable number of separate clusters within *S. grandis* (Supplementary Fig. S2), where one pure cluster represented individuals from populations 7 and 8, while 9 out of 13 specimens of population 3 and 4 constituted the second pure group. The rest of *S. grandis* specimens were admixed between these pure clusters. Lastly, based on the PCoA individuals of *S. krylovii* were clustered into the two main groups representing population 8 from Kyrgyzstan and the remaining populations from Russia (Fig. 5d). Although few specimens of population 1 were genetically more distant to the other individuals, the second axis of PCoA explained only 5.4% of the total genetic divergence. The pairwise *F*st values also supported the division within *S. krylovii* into two groups indicating the strong differentiation of population 8 (*F*st in a range of 0.37–0.46) and a moderate or near to moderate differentiation among the remaining populations (*F*st in a range of 0.06–0.19). Similarly, the STRUCTURE analysis revealed two genetic clusters (Supplementary Fig. S2) that described the population structure the best: the first one represented population 8 from Kyrgyzstan, while the second cluster comprised populations from Russia.

### Divergence-time estimation

**Fig. 6:**
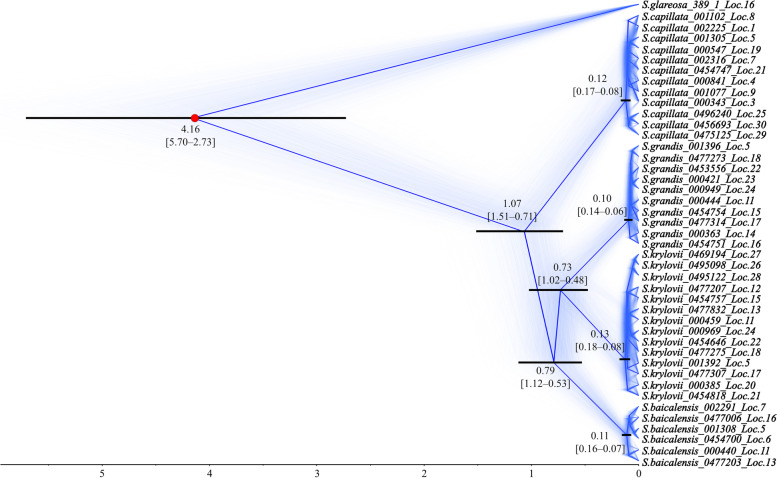
Phylogeny and divergence date estimates inferred by SNAPP. Blue coloured trees represent the most probable topology. Numbers at each node represent mean ages of divergence time estimates and the 95% HPD intervals (in the brackets). The black rectangles on the nodes indicate the 95% HPD intervals of the estimated posterior distributions of the divergence times. The red circle indicates the presumed divergence time split set as a reference. The Bayesian posterior probabilities were 1.00 for the nodes with the shown 95% HPD intervals. The scale shows divergence time in Mya

The SNAPP phylogeny based on 2717 SNP markers among pure individuals revealed largely the same topology as the UPGMA dendrogram, except for the pair *S. grandis* and *S. krylovii* that were grouped together, while *S. baicalensis* was a sister taxon (Fig. 6 and Supplementary Fig. S3). The result suggests that the potential split between *S. capillata* and three species, namely, *S. grandis*, *S. krylovii* and *S. baicalensis*, took place approximately 1.07 Mya with the 95% Highest Posterior Density interval (HPD) of 1.51–0.71 Mya. The most recent common ancestor for *S. grandis*, *S. krylovii* and *S. baicalensis* was inferred to be 0.79 Mya (95% HPD: 1.12–0.53 Mya), whereas the lowest divergence time of 0.73 Mya was registered for *S. grandis* and *S. krylovii* (95% HPD: 1.02–0.48 Mya). The chronogram also indicates that diversification within *S. capillata*, *S. krylovii* and *S. baicalensis* started at relatively the same time, ca. 130–114 kya (95% HPD: 181–74 kya), while *S. grandis* was established to be the youngest species (96 kya, 95% HPD: 137–63 kya). Lastly, although the topologies within the species had large uncertainty, some nodes had comparatively high Bayesian posterior probabilities (BPP ≥ 0.80; Supplementary Fig. S3). In particular, individuals of *S. capillata* from Kyrgyzstan started to differentiate 97 kya (95% HPD: 137–58 kya), while the well-supported split (BPP of 0.92) between specimens from localities 5 (Russia, the Republic of Khakassia) and 19 (Russia, the Republic of Buryatia) took place 86 kya (95% HPD: 122–53 kya). The most recent common ancestor for individuals of *S. krylovii* from Kyrgyzstan was inferred to be 62 kya (95% HPD: 94–35 kya), whereas specimens from localities 22 (Mongolia) and 24 (Russia, Zabaykalsky Krai) had the potential split 68 kya (95% HPD: 99–40 kya). Interestingly, specimens of *S. baicalensis* from localities 11 and 13 (both from Russia, the West shore of Lake Baikal) and individuals from the remaining localities had nearly the same divergence times of 97 kya (95% HPD: 140–60 kya) and 93 kya (95% HPD: 132–58 kya), respectively. Additionally, the potential split between populations of *S. grandis* from the Republic of Khakassia (locality 5) and the Republic of Buryatia (locality 18) took place 65 kya (95% HPD: 91–39 kya), while the well-supported split (BPP of 0.95) between specimens from localities 23 and 24 (both from Russia, Zabaykalsky Krai) took place 58 kya (95% HPD: 86–34 kya).

### Morpho-molecular analysis

**Fig. 7:**
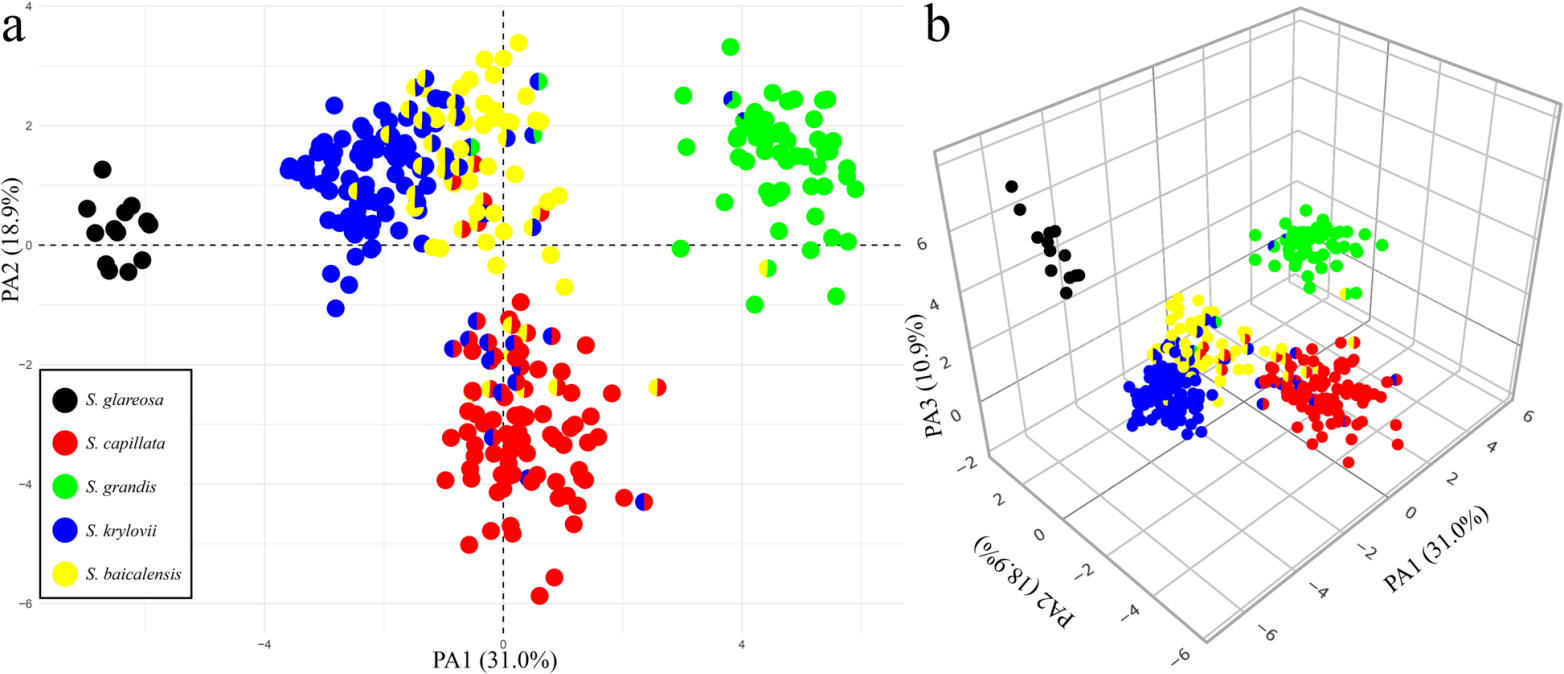
The factor analysis of mixed data performed on 17 quantitative and six qualitative characters of the five examined species of *Stipa*. **a** Plot of the two principal axes. **b** Plot of the three principal axes. The pie charts represent the proportions of membership established by fastSTRUCTURE for the best K = 5

As non-parametric Spearman correlation coefficients did not demonstrate any strong correlation (> 0.90) between the measured variables (Supplementary Fig. S4), we retained all morphological characters (Table 3) for a factor analysis of mixed data (FAMD) and analyses of notch plots. Subsequently, to investigate if the observed phenotypes are congruent with molecular data, we supplemented the result of the FAMD analysis with the genetic clusters inferred by fastSTRUCTURE for the best K = 5. As a result, the FAMD revealed five markedly differentiated groups (Fig. 7) of operational taxonomic units (OTUs) in accordance with the detected clusters using the SNP data (Figs. 2, 3 and 4). The first four dimensions explained 31.0, 18.9, 10.9 and 8.1% of the total variability, respectively. The first three axes are mainly composed by the contribution of 11 quantitative and four qualitative variables (Supplementary Table S4).

Importantly, due to having the genetic clusters assigned by fastSTRUCTURE, the two-dimensional plot revealed the slight overlapping of OTUs belonging to the pure species *S. baicalensis* and *S. krylovii*, whereas OTUs representing the admixed specimens *S. baicalensis* × *S. krylovii* were present in both clouds of the parental taxa (Fig. 7a). Furthermore, hybrids *S. capillata* × *S. baicalensis* were also present in both clouds of the pure species. On the other hand, all the admixed individuals *S. capillata* × *S. krylovii* were grouped together with only one parental species, *S. capillata.* Interestingly, hybrids between *S. grandis* and *S. krylovii* were mainly grouped together with OTUs of *S. baicalensis* with an exception of the first- and the second-generation backcrosses. The only hybrid detected between *S. grandis* and *S. baicalensis* was clustered together with the pure individuals of the former taxon. A more clear dispersal of the pure species can be seen in the three-dimensional plot, where differences between the studied species are explained by the third principal axis (Fig. 7b; an interactive version of the plot available at https://plot.ly/~eugenebayahmetov/42/). Additionally, all combinations of two axes plots for the four dimensions are present in Supplementary Fig. S5.

The result of FAMD and notch plots of the quantitative variables demonstrated that the studied species can be differentiated morphologically mainly based on the length of the lower segment of the awn (Col1L), the distance from the end of the dorsal line of hairs to the top of the lemma (DDL), the length of the anthecium (AL), the length of the middle segment of the awn (Col2L), the length of the callus (CL), the length of ligules of the internal vegetative shoots (LigIV), the length of ligules of the middle cauline leaves (LigC) and the length of hairs on the top of the lemma (LHTA). In addition, the length of hairs on the lower segment of the awn (HLCol1), the length of hairs on the middle segment of the awn (HLCol2) and the length of the callus base (CBL) can aid to distinguish *S. glareosa* from the remaining taxa (Supplementary Fig. S6). For instance, the notch plot of Col1L showed significant differences between means and strong evidence of differing medians within the pure species except the pair *S. baicalensis* and *S. capillata*, while the hybrid individuals had mostly intermediate positions between the parental species except specimens of *S. capillata* × *S. baicalensis* that were significantly different from the samples of the pure taxa. The differences across all quantitative variables between individuals of the pure species and the hybrids can be better evaluated in the interactive box plots presented in Supplementary File 1.

Among the qualitative variables, the main contribution to the axes of the FAMD had the abaxial surface of vegetative leaves (AbSVL), the type of hairs on the top of the anthecium (HTTA), the type of the awn geniculation (AG) and the presence of hairs below nodes (PHBN) (Supplementary Table S4). For instance, vegetative leaves with prickles were common in *S. glareosa* (all samples), *S. capillata* (61 out of 66 samples), *S. capillata* × *S. krylovii* (12 out of 14 samples), *S. capillata* × *S. baicalensis* (10 out of 14 samples), less frequent in *S. grandis* (2 out of 54 samples), *S. krylovii* (5 out of 81 samples), *S. baicalensis* × *S. krylovii* (2 out of 14 samples) and totally absent in *S. baicalensis*, *S. grandis* × *S. krylovii* and *S. grandis* × *S. baicalensis* (Supplementary Fig. S7).

Thus, based on the results of the molecular analyses and the FAMD combining both phenotypic and SNP data, we were able to differentiate the pure species and the hybrid individuals at the morphological level. We established that using the traditional identification keys [16, 43–45] 71 out of 302 specimens had been misidentified, mostly due to their hybrid nature (Supplementary Table S5). In particular, 47 samples previously identified as pure species appeared to be hybrids. The remaining 24 specimens earlier were classified either as hybrids (15 samples) or misleadingly assigned to *S. baicalensis* (9 samples)*.* Interestingly, in the latter case, all individuals were previously reported from the northeastern part of Kazakhstan [46].

In general, *S. baicalensis* was the most problematic species for taxonomic identification comprising 54 doubtful samples. Specifically, the above-mentioned specimens from Kazakhstan genetically were proven to be pure *S. capillata*, 10 specimens appeared to be hybrids *S. capillata* × *S. baicalensis*, 8 were *S. baicalensis* × *S. krylovii* and 10 were hybrids between *S. capillata* and *S. krylovii*. On the other hand, specimens previously identified as hybrids *S. baicalensis* × *S. krylovii* (3 samples) and *S. grandis × S. krylovii* (2 samples) were genetically assigned to the pure species *S. baicalensis.* Oppositely, specimens morphologically identified as *S. krylovii* (8 samples), *S. grandis* (1 sample) and *S. capillata* (1 sample) appeared to be hybrids *S. baicalensis* × *S. krylovii* (6 samples) and *S. capillata* × *S. baicalensis* (2 samples), *S. grandis* × *S. baicalensis* and *S. capillata* × *S. baicalensis*, respectively. Additionally, one specimen *S. capillata* × *S. grandis* was proven to be *S. capillata* × *S. baicalensis*. At last, one individual *S. capillata* × *S. baicalensis* was genetically verified to be the pure species *S. capillata*.

The remaining doubtful samples morphologically were either misleadingly assigned as the pure species or as taxa of hybrid nature. For instance, 4 specimens of *S. capillata* were hybrids between *S. capillata* and *S. krylovii*, while specimens of *S. grandis* (2 samples) and *S. krylovii* (2 samples) were shown to be genetically admixed as *S. grandis* × *S. krylovii*. In opposite, 9 individuals identified as *S. capillata* × *S. krylovii* appeared to be the pure species *S. capillata*. Interestingly, only 5 out of 52 hybrid taxa were properly identified based on morphology including *S. baicalensis* × *S. krylovii* (3 samples) and *S. grandis* × *S. krylovii* (2 samples). The only one taxon that had not any questionable individuals was *S. glareosa* representing the section *Smirnovia.*

## Discussion

The current understanding of taxonomy and species limits in *Stipa* is still largely based on morphological characters. Our study highlights the necessity of using molecular tools to properly identify taxa and detect processes underlying speciation. This is of particular relevance in hybrid zones where ongoing hybridisation and introgression may lead admixed individuals to phenotypes similar to one of the parental species, complicating identification of such taxa using morphological characters alone. Furthermore, integrative studies suggest that apparently intermediate phenotypes between two species are not necessarily hybrids [47]. On the other hand, as indicated in the present work, although some interspecific hybrids have intermediate characters between parental taxa, their phenotypic traits can also overlap with non-parental species leading to misidentification. Given the circumstances, here we utilised an integrative approach combining genome-wide data and morphology to delimitate species and ascertain the extent of hybridisation in feather grasses.

The study clearly illustrates that a molecular-based analysis, e.g., such as fastSTRUCTURE, combined with a factor analysis of mixed data, utilising both quantitative and qualitative variables, can largely resolve the problem with species identification in the face of ongoing hybridisation and introgression. Primarily, such an approach allows to visualise and easily trace if the observed phenotypes are congruent with molecular data. Besides that, this approach may aid in selecting a set of traits that can be useful for species identification in the field. Our findings clearly demonstrate that the studied individuals can be clustered into five species groups representing *S. capillata*, *S. baicalensis*, *S. glareosa*, *S. grandis* and *S. krylovii*. Thus, here we found no evidence that *S. baicalensis* is of hybrid origin from *S. grandis* × *S. krylovii* or *S. capillata* × *S. krylovii* but is instead a genetically distinct species. The general branching of the phylogenetic trees is in good agreement with the current taxonomic classification. In particular, a representative of the section *Smirnovia*, *S. glareosa*, was genetically distant to the remaining species from the section *Leiostipa*. Nevertheless, our result contradicts a previous research, where *S. capillata*, *S. krylovii* and *S. baicalensis* represented one clade and *S. grandis* was a sister taxon [19], while here, such a sister taxon was *S. capillata.* The current result is likely more accurate due to applying several thousand SNPs across the genome in comparison to only one nuclear locus in the above-mentioned study. Additionally, we demonstrated that the potential split between *S. capillata* and the remaining representatives of *Leiostipa* took place approximately 1.07 Mya, which is similar to our previous estimate of 1.73 Mya based on the nucleolar organising regions [48]. Here, we also reported for the first time that diversification within species *S. capillata*, *S. baicalensis*, *S. krylovii* and *S. grandis* started ca. 130–96 kya (95% HPD: 181–63 kya). These ages may correspond to the potential window of time between the Last Interglacial period, which began around 130 kya, and the Last Glacial Period (LGP), which started about 110 kya. Thus, the observed pattern is similar to dispersing events reported for different taxa across the plant kingdom [49, 50] suggesting climatic changes as a feasible factor in the current distribution of feather grasses. Of note, due to the divergence times that were inferred in SNAPP, which uses the multi-species coalescent model ignoring possible introgression, the confidence may be exaggerated. On the other hand, although introgression does cause biased errors in coalescent-based species tree inference [51–53], it should not affect the estimates in the present study, since all admixed individuals were excluded from the analysis.

Importantly, the results of molecular analyses were congruent and provided evidence for the existence of hybridisation between pairs *S. capillata* × *S. baicalensis*, *S. capillata* × *S. krylovii*, *S. grandis × S. krylovii*, *S. grandis × S. baicalensis* and *S. baicalensis* × *S. krylovii*. The presence of F2 or more advanced hybrid generations suggests that F1 individuals are able to reproduce further. This observation is in agreement with our previous findings on hybridisation within the genus [32, 33], where a direct approach proved that a hybrid taxon *S. heptapotamica* produces fertile pollen grains and is capable of backcrossing to primarily one parental species [33]. Indeed, here we detected backcrosses *S. baicalensis* × *S. krylovii* and *S. grandis × S. krylovii* to their former parental species. Moreover, the analysis of BABA/ABBA patterns among species revealed signs of past introgression between *S. baicalensis* and *S. krylovii*, *S. baicalensis* and *S. grandis*, *S. krylovii* and *S. grandis.* Taking into account the diversification times of these species, we can hypothesise that if such a gene flow occurred it was relatively recent in evolutionary terms and seemingly still present between *S. baicalensis* and *S. krylovii*, as well as in pair *S. grandis* and *S. krylovii.* Nevertheless, we treat our BABA/ABBA analysis with caution due to such a test originally being applied in human studies whose genome is available at the chromosome level and the number of SNPs was remarkably higher than used here. Thus, we intend to reassess the past gene flow within these taxa when a more continuous genome will be obtained. Additionally, although here we did not detect any backcrosses between *S. baicalensis* and *S. grandis*, it cannot be excluded that such a combination exists in nature, especially since both species are mostly common in Mongolia and China; however specimens from there were not presented in the study. Lastly, our study revealed only unidirectional backcrossing of hybrid taxa either to *S. baicalensis* or *S. grandis*, but not to *S. krylovii*. Similarly, due to the relatively small sample size and the limited number of localities used here, we cannot draw any reliable conclusions concerning possible barriers to gene flow to the latter taxon.

According to our results, the populations’ expansion seemingly started during the LGP. Nonetheless, the assessment of genetic structures revealed signs of contemporary gene flow between populations across all species, despite significant geographical distances between some of them. For instance, populations of *S. baicalensis*, *S. capillata* and *S. grandis* from the eastern part of Khakassia and the southwestern area of Buryatia represented either one genetic cluster or were admixed between two. Among potential explanations are shifting their distributions in response to climate change, or seasonal migration of wild animals and livestock grazing. While we currently do not possess enough data to verify the first assumption, seeds of feather grasses usually are spread naturally by wind or water. On the other hand, they also can be frequently dispersed by the wool of mammals including, e.g., sheep, goats and horses [16, 18]. Although this study was not intended to explore population differences in detail, we believe that our findings regarding gene flow merit further studies in order to better understand the intraspecific variation and relationships among populations as well as to discuss potential consequences of such events.

Our results also illustrated a complex association between species at the morphological level. There are usually few phenotypic characters differentiating hybridising species and these characters are often functionally or developmentally correlated [54]. In the present case, although the studied species had a set of distinctive characters, the current identification keys do not provide a solution on distinguishing admixed individuals in the section *Leiostipa*. As a result, only 5 out of 52 hybrid taxa were properly identified based on morphology. Furthermore, the results demonstrated that the most problematic taxon is *S. baicalensis*, which was frequently misidentified either with *S. capillata* or a cross *S. capillata* × *S. krylovii*, while a few individuals were misleadingly assigned to *S. grandis* × *S. krylovii*. Additionally, several hybrids between *S. baicalensis* and *S. capillata* were determined as pure *S. krylovii*. Therefore, we believe that the identification keys should be revised in order to properly delimitate pure species and propose a taxonomic treatment for the hybrid taxa identified in the study. Moreover, for a more comprehensive morphological assessment we suggest using scanning electron microscopy that can assist in finding unique ultrastructures among pure and admixed individuals.

Although here we highlight that morphological characters alone cannot be used to properly identify hybrids and backcrossed individuals in the field, it is a common issue in plants rather than an exception in feather grasses. For instance, in a study on three species of willows it was shown that based on phenotype only 5% of specimens were classified as introgressed individuals, which was much less than the 19% detected using SNP data [55]. Another research on tropical trees demonstrated that even limited genomic sampling, when combined with morphology and geography, can greatly improve estimates of species diversity for clades where hybridisation contributes to taxonomic difficulties [56]. Recently, an investigation on several pine species using SNP data derived from the DArTseq pipeline revealed that one species, previously considered of hybrid origin, is a genetically distinct species and provided insights into the challenges of solely using morphological traits when identifying taxa with cryptic hybridisation and variable morphology [57].

In grasses, hybridisation and introgression phenomena are still mainly studied in crop species, e.g., rice [58], wheat [59] and sugarcane [60]. To date, we have detected hybrids not only between genetically closely related species [33], but also among genetically distant *Stipa* taxa [13, 34]. The results present here and our previous findings helps us to shift toward thinking of the *Stipa* phylogeny as reticulate webs rather than a strictly bifurcating tree. Nonetheless, studying hybridisation in feather grasses is not only of particular interest to plant taxonomists. The presence of parental species, multiple generation hybrids and backcrosses in different proportions in a hybrid zone may indicate renewed sympatry providing important data for studying species boundaries and patterns of speciation [61]. From this point of view, *Stipa* may be a suitable genus to study these phenomena. Despite the increasing interest in feather grasses at the molecular level [19, 20, 48, 62–65], there is still a lack of substantial knowledge regarding, e.g., chromosome numbers of admixed and pure taxa in hybrid zones, fertility of pollen grains in F1 and later generation hybrids and backcrosses and genomic information related to specific loci contributing to reproductive barriers. We believe that only an integrative approach combining the aforesaid data can properly interpret evolutionary patterns and processes in feather grasses.

## Conclusions

In the current study we revealed a complex taxonomic issue in feather grasses with variable morphology exhibited due to extensive hybridisation. Based on SNPs derived from genome-wide genotyping we detected five genetic groups representing separate morphospecies and showed that *S. baicalensis* is a genetically distinct species instead of a taxon of hybrid origin as it was previously hypothesised. We demonstrated the presence of F1 hybrids between *S. capillata × S. baicalensis*, *S. capillata × S. krylovii*, *S. grandis × S. krylovii*, *S. grandis × S. baicalensis*, *S. baicalensis × S. krylovii* and F2 individuals in *S. capillata × S. krylovii*, *S. grandis × S. krylovii*, *S. baicalensis × S. krylovii* indicating low levels of reproductive isolation in these species. We also discovered a few backcrosses *S. baicalensis* × *S. krylovii* and *S. grandis* × *S. krylovii* to their former parental species suggesting possible introgression among the taxa.

Furthermore, we detected reticulation events between *S. baicalensis* and *S. krylovii*, *S. baicalensis* and *S. grandis*, *S. krylovii* and *S. grandis.* On the other hand, we revealed signs of contemporary gene flow between populations of the species from the section *Leiostipa*. Another important outcome of the research is divergence date estimates inferred at the species and population levels. Here we deduce that diversification within the studied species started ca. 130–96 kya and hypothesise that climatic changes during the LGP were a driving force behind the current distribution of feather grasses. Importantly, here we also emphasise the usefulness of applying integrative approaches combining molecular and morphological data to delimitate species and detect hybridisation and introgression events in feather grasses. Finally, we conclude that *Stipa* may be a suitable genus to study these phenomena.

## Methods

### Plant material

In total, 302 fully developed *Stipa* samples were used for molecular and morphological studies. We gathered individuals representing the section *Leiostipa* (*S. baicalensis*, *S. capillata*, *S. grandis* and *S. krylovii*) from localities where these taxa grow together as well as from areas where they grow separately from each other (Fig. 1, Supplementary Table S1). Additionally, we included two populations of *S. baicalensis* previously reported from the northeastern part of Kazakhstan [46] and 13 specimens of *S. glareosa* belonging to the section *Smirnovia* that were found in localities 10, 12, 13 and 16. All voucher specimens used in the study are preserved at TK and KRA. All maps were visualised using ArcGIS Pro 2.7.1 (ESRI, Redlands, USA). The species distribution ranges were established based on the revision of herbarium specimens preserved at AA, ALTB, B, BM, BRNU, COLO, E, FR, FRU, G, GAT, GFW, GOET, IFP, K, KAS, KFTA, KHOR, KRA, KRAM, KUZ, L, LE, LECB, M, MO, MSB, MW, NY, P, PE, PR, PRC, TAD, TASH, TK, UPS, W, WA, WU and Z.

### DNA extraction, amplification and DArT sequencing

This section was performed according to the previously reported procedures [34]. In brief, genomic DNA was isolated from dried leaf tissues using a Genomic Mini AX Plant Kit (A&A Biotechnology, Poland) and sent to Diversity Arrays Technology Pty Ltd. (Canberra, Australia) for the following genome complexity reduction using restriction enzymes and high-throughput polymorphism detection [66].

All DNA samples were processed in digestion/ligation reactions as described previously [66], but replacing a single PstI-compatible adaptor with two different adaptors corresponding to two different restriction enzyme overhangs. The PstI-compatible adapter was designed to include Illumina flowcell attachment sequence, sequencing primer sequence and “staggered”, varying length barcode region, similar to the sequence previously reported [37]. The reverse adapter contained a flowcell attachment region and MseI-compatible overhang sequence. Only “mixed fragments” (PstI-MseI) were effectively amplified by PCR using an initial denaturation step of 94 °C for 1 min, followed by 30 cycles with the following temperature profile: denaturation at 94 °C for 20 s, annealing at 58 °C for 30 s and extension at 72 °C for 45 s, with an additional final extension at 72 °C for 7 min. After PCR equimolar amounts of amplification products from each sample of the 96-well microtiter plate were bulked and applied to c-Bot (Illumina, USA) bridge PCR followed by sequencing on Hiseq2500 (Illumina, USA). The single read sequencing was performed for 77 cycles.

Sequences generated from each lane were processed using proprietary DArT analytical pipelines. In the primary pipeline, the fastq files were first processed to filter away poor quality sequences, applying more stringent selection criteria to the barcode region compared to the rest of the sequence. In that way the assignments of the sequences to specific samples carried in the “barcode split” step were reliable. Approximately 2.5 mln sequences per barcode/sample were identified and used in marker calling.

### DArTseq data analysis

For the downstream analyses, we applied co-dominant single nucleotide polymorphism (SNP) markers processed in the R-package dartR v.1.5.5 [67] with the following parameters: (1) a scoring reproducibility of 100%, (2) SNP loci with read depth < 5 or > 50 were removed, (3) at least 95% loci called (the respective DNA fragment had been identified in greater than 95% of all individuals), (4) monomorphic loci were removed, (5) SNPs that shared secondaries (had more than one sequence tag represented in the dataset) were randomly filtered out to keep only one random sequence tag.

### DNA-based species delimitation

Five approaches were used to analyse the genetic structure at the species level: (1) Unweighted Pair Group Method with Arithmetic Mean (UPGMA), (2) fastSTRUCTURE analysis, (3) Principal Coordinates Analysis (PCoA), (4) NewHybrids analysis, (5) calculation of the *f*_4_ statistic. In addition, to assess the genetic differentiation at the population level within *S. baicalensis*, *S. capillata*, *S. grandis* and *S. krylovii* we performed PCoA and STRUCTURE analyses and calculated *F*_ST_. Furthermore, we used SNAPP to estimate divergence times within the studied species and populations.

Firstly, a UPGMA cluster analysis based on Jaccard’s distance matrix was performed using R-packages dartR and visualised with stats v.3.6.2 [68]. Next, the genetic structure was investigated using fastSTRUCTURE v.1.0, which implements the Bayesian clustering algorithm STRUCTURE, assuming Hardy-Weinberg equilibrium between alleles, in a fast and resource-efficient manner [69]. A number of clusters (K-values) ranging from 1 to 10 were tested using the default parameters with ten replicate runs per dataset. The most likely K-value was estimated with the best choice function implemented in fastSTRUCTURE. The output matrices for the best K-values were reordered and plotted using the R package pophelperShiny v.2.1.0 [70]. We applied the threshold of 0.10 < q < 0.90 as the most widely utilised measure for the assessment of hybridisation [71, 72] with q-values > 0.9 being pure species and 0.45 < q < 0.55 being F1 hybrids, while first- and second-generation backcrosses with one parent were considered at q-values 0.25 and 0.125, respectively [73]. Then, a PCoA on a Euclidean distance matrix was performed using the R-package dartR and visualised with ggplot2 v.3.3.0 [74] to show the first two components and plotly v.4.9.2 [75] to illustrate the first three components.

### Hybrid generation identification

Next, to assign individuals to a genetic category (pure, hybrid F1 or F2, backcross hybrid) based on their multilocus genotypes, we performed analyses using a Bayesian model-based clustering method implemented in NewHybrids v.1.1 [76] via the R package dartR. The program utilises Markov chain Monte Carlo (MCMC) simulations to compute the posterior probability of an individual belonging to pre-defined categories comparing two parental genotypes at a time. Thus, we used five data sets representing parental species and their hybrids defined by the UPGMA, fastSTRUCTURE and PCoA outputs from the previous steps. Specifically, we had the following datasets: (1) *S. baicalensis*, *S. krylovii* and the admixed individuals between the parental species; (2) *S. baicalensis*, *S. capillata* and the admixed individuals; (3) *S. grandis*, *S. krylovii* and the admixed individuals; (4) *S. capillata*, *S. krylovii* and the admixed individuals; (5) *S. baicalensis*, *S. grandis* and the admixed individuals. Due to limitations of NewHybrids, only 200 loci were used per run. In the beginning, the first 200 loci ranked on information content (option “AvgPIC”) were chosen via dartR. Then, to ensure the convergence of the algorithm, we used ten different 200-SNP subsets selected in random (option “random”). The Jeffreys prior was used for θ and π and a burn-in of 10,000 MCMC generations followed by 10,000 post burn-in sweeps. The resulting posterior probabilities were calculated based on 11 runs and a probability threshold > 0.8 was set for the assignment into a genetic category. The calculated posterior probabilities for the assigned categories were visualised using the R package pophelperShiny.

### Testing for introgression

Further, to calculate the *f*_4_ statistic we retained only pure individuals determined via the UPGMA, fastSTRUCTURE and PCoA analyses. All filtering steps were conducted using the R-package dartR with the above-mentioned sequence. Next, the processed data was converted into the EIGENSTRAT format using the R-package dartR. The subsequent calculation of *f*_4_ statistics was performed in ADMIXTOOLS v. 7.0.1 [77] using the R package admixr v.0.9.1 [78]. In brief, the *f*_4_ statistic [79] is similar to the *D* statistic [80, 81] and measures the average correlation in allele frequency differences between four populations, e.g., A, B, C and D [82]. If a divergent outgroup is provided as population A, we can test for gene flow between B and C (if the statistic is negative and Z-score < − 3) or B and D (if the statistic is positive and Z-score > 3). Due to such a test originally being applied to continuous genome-wide data, it is important to mention some limitations of the analysis while working with non-model organisms. If a draft genome is available, it is possible to specify positions of SNPs along contigs or scaffolds by a special parameter “blockname” implemented in ADMIXTOOLS. Nonetheless, the package currently does not support more than 600 contigs/scaffolds [83] decreasing the potential number of used SNPs for very fragmented genomes. That was the case of *Stipa*, where only one draft genome comprising 5931 contigs is currently available [48]. Additionally, SNPs positions and genetic distances are used for a block jackknife method to test for a significant deviation from the null expectation of the *f*_4_ statistic. Thus, in the absence of a reference genome or the presence of a very fragmented genome the *f*_4_/*D* statistics should be treated with caution. Here we consider signatures of past gene flow events only if BABA or ABBA patterns are greater than 50. All possible four-species combinations were tested, while one species, *S. glareosa*, was selected as an outgroup (A) for all runs.

### Population differentiation

To perform PCoA and STRUCTURE analyses and calculate *F*_ST_ at the population level we retained only pure species and kept populations with more than three individuals per population. To maintain as many populations as possible, we merged several individuals growing relatively close to each other (Table 2 and Supplementary Table S1). All filtering steps were conducted using the R-package dartR with the above-mentioned sequence. PCoA analyses were also performed according to the aforesaid flow. Next, we used STRUCTURE v.2.3.4 [84] instead of fastSTRUCTURE due to the former software being markedly superior to the latter one under weak population differentiation [85]. To overcome the relatively slow speed of the analysis we applied parallel computing using StrAuto v.1.0 [86]. Five replicate runs were performed for each number of clusters (*K*) from one to ten with a burn-in of 50,000 iterations followed by 500,000 MCMC iterations. The optimal *K* value was identified based on Evanno’s method of ΔK statistics [87] as implemented in Structure Harvester [88]. The calculated posterior probabilities for the assigned categories were visualised using the R package pophelperShiny. Then, the fixation index *F*st was calculated using the R-package dartR with 1000 bootstraps to obtain *p*-values. This measure assesses genetic differentiation among populations, where values of 0.00–0.05 indicate low differentiation, 0.05–0.15 indicate moderate differentiation, while *F*st > 0.15 indicates high levels of differentiation [89].

**Table 2:** Analysed populations

Species	Populations and their localities according to Fig. 1 and Supplementary Table S1	Number of individuals per population
*S. baicalensis*	Population 1 (localities 5, 6 and 7)	5
	Population 2 (locality 11)	8
	Population 3 (locality 13)	15
	Population 4 (locality 16)	8
*S. capillata*	Population 1 (locality 1)	3
	Population 2 (locality 3)	7
	Population 3 (locality 4)	7
	Population 4 (locality 5)	6
	Population 5 (locality 8)	19
	Population 6 (locality 9)	9
	Population 7 (locality 19)	6
	Population 8 (locality 21)	5
	Population 9 (localities 25, 29 and 30)	4
*S. grandis*	Population 1 (locality 5)	3
	Population 2 (locality 11)	7
	Population 3 (locality 14)	7
	Population 4 (locality 16)	6
	Population 5 (localities 15 and 17)	8
	Population 6 (locality 18)	8
	Population 7 (locality 23)	7
	Population 8 (locality 24)	7
*S. krylovii*	Population 1 (locality 11)	15
	Population 2 (locality 15)	7
	Population 3 (locality 17)	6
	Population 4 (locality 18)	8
	Population 5 (locality 20)	7
	Population 6 (locality 21)	10
	Population 7 (locality 24)	6
	Population 8 (localities 26, 27 and 28)	20

### Divergence-time estimation

Finally, to estimate divergence times between the studied taxa, we retained only one pure individual per species and population using dartR and followed the above-mentioned sequence of filtering steps with an exception of the called loci (100% instead of 95%). Then, the SNP data was converted to the Nexus format using dartR. Subsequently, we created an XML file using the SNAPP-specific template provided in Stange et al., 2018. SNAPP v.1.5.1 [90] utilises the multi-species coalescent approach and is well-suited for analyses of genome-wide data deducing the species tree and divergence times directly from SNPs [91]. We applied one time calibration, setting a log-normal distribution with a mean of 4.39 Mya and a standard deviation (SD) of 0.18 for the split between *S. glareosa* (section *Smirnovia*) and *S. capillata* (section *Leiostipa*) as it was inferred in our previous study [48]. The analysis was performed three times independently, 1.25 million MCMC generations for each run using BEAST2 v.2.6.3 [92]. Tracer v.1.7.1 [93] was used to visually check the combined log file regarding Effective Sample Size (ESS) values. As all ESSs exceeded 200, we combined tree files using LogCombiner v.2.6.3 (a part of the BEAST package) with the first 10% discarded as burn-in from each run. The final maximum clade credibility tree was summarised in TreeAnnotator v.2.6.3 (a part of the BEAST package). Lately, we visualised a pattern across all of the posterior trees via DensiTree v.2.01 [94], while FigTree v.1.4.4 [95] was used to inspect the Bayesian posterior probabilities and the 95% credibility intervals of the final tree.

### Morphological analysis

A total of 302 specimens were examined under a light microscope SMZ800 (Nikon, Japan) across the 17 most informative quantitative and six qualitative morphological characters commonly used in keys and taxonomic descriptions of *Stipa* (Table 3). Firstly, the Shapiro-Wilk test was used in the R-package MVN v.5.8 [96] to assess the normality of the distribution of each characteristic. Secondly, the non-parametric Spearman’s correlation test was applied using R-packages stats and Hmisc v.4.3–1 [97] to examine relations between the studied characters. The combined correlogram with the significance test was visualised using the R-package corrplot v.0.84 [98].

**Table 3:** Morphological characters used in the present study

Character	Abbreviation
**Quantitative characters (mm)**	
Width of blades of vegetative shoots	WVS
Length of ligules of the middle cauline leaves	LigC
Length of ligules of the internal vegetative shoots	LigIV
Length of the lower glume	LG
Length of the anthecium	AL
Length of the callus	CL
Length of the callus base	CBL
Length of hairs on the dorsal line on the lemma	LHD
Length of hairs on the ventral line on the lemma	LHV
Distance from the end of the dorsal line of hairs to the top of the lemma	DDL
Distance from the end of the ventral line of hairs to the top of the lemma	DVL
Length of hairs on the top of the lemma	LHTA
Length of the lower segment of the awn	Col1L
Length of the middle segment of the awn	Col2L
Length of the seta	SL
Length of hairs on the lower segment of the awn	HLCol1
Length of hairs on the middle segment of the awn	HLCol2
**Qualitative characters**	
Character of the abaxial surface of vegetative leaves (glabrous, with prickles)	AbSVL
Character of the adaxial surface of vegetative leaves (short hairs, long hairs, mixed)	AdSVL
Type of the awn geniculation (single, double)	AG
Character of nodes (glabrous, with hairs)	CN
Type of hairs on the top of the anthecium (glabrous, poor developed, well developed)	HTTA
Presence of hairs below nodes (glabrous, with hairs)	PHBN

Next, a Factor Analysis of Mixed Data (FAMD) [99] was accomplished using the R-package FactoMineR v.2.3 [100] to characterise the variation within and among groups of taxa without a priori taxonomic classification and to extract the variables that best identified them. The number of principal components used in the analysis was chosen based on Cattell’s scree test [101]. R-packages factoextra v.1.0.6 [102] and plotly were used to visualise the first two and the first three principal components, respectively. Subsequently, the plots were supplemented with the result of the fastSTRUCTURE analysis for the best K = 5.

Additionally, to evaluate distributional relationships between each response variable and the studied taxa, notch plots and interactive box plots were created using R-packages ggplot2 v.3.3.0 and plotly v.4.9.2, respectively. The notched box plots display a confidence interval around the median, which is normally based on the median ± 1.57 × interquartile range/square root of n. According to this graphical method for data analysis, if the notches of the two boxes do not overlap, there is “strong evidence” (95% confidence) that their medians differ. In addition, to reveal significant differences between means of particular characters across all examined taxa the nonparametric Kruskal-Wallis test followed by the post-hoc Wilcoxon test with Bonferroni correction were performed using the R-package stats v.3.6.2.

## Supplementary Information

**Additional file 1.** Interactive box plots.

**Additional file 2: Supplementary Table S1**. List of samples used in the study. **Table S2**. Average posterior probabilities inferred in NewHybrids for first (F1) and second (F2) generation hybrids and backcrosses (F1xP1 and F1xP2). **Table S3**. Pairwise *F*st values for population differentiation across the four studied species. *F*st > 0.15 indicating high levels of differentiation are in bold type. **Table S4**. Contribution (%) by dimension of each character (abbreviations according to Table 3) in FAMD. The first five characters contributing the most are in bold type. Abbreviations of the qualitative variables and their contributions to the principal axes are underlined. **Table S5**. The assigned species names based on morphological and molecular data. Mismatches are shown in bold type. **Supplementary Figure S1** Venn diagram representing polymorphic SNPs among four pure *Stipa* species. The admixed individuals and *S. glareosa*, which did not show patterns of hybridisation, were omitted in the metric’s calculation. **Supplementary Figure S2** Delta K values calculated by Evanno’s method across four species. (a) *S. baicalensis*. (b) *S. capillata.* (c) *S. grandis.* (d) *S. krylovii.* **Supplementary Figure S3** Phylogeny (at the top) and divergence date estimates at the species level (on the bottom) inferred by SNAPP. The scale shows divergence time in Mya. The red circles indicate nodes with the Bayesian posterior probabilities (BPP) > 0.8. The lower-case letters refer to the embedded table containing data regarding the exact estimates of the divergence times (in kya), BPPs and 95% HPD intervals. **Supplementary Figure S4** Correlation matrix of the studied morphological characters (abbreviations according to Table 3). Colour intensity and the size of the circle are proportional to the correlation coefficients (displayed in the circle). Positive correlations are blue while negative are red. All *p*-values of Pearson correlations were < 0.01. **Supplementary Figure S5** Factor analysis of mixed data performed on 17 quantitative and six qualitative characters of the five examined species of *Stipa*. (a) Plot of the principal axes one and two. (b) Plot of the principal axes one and three. (c) Plot of the principal axes one and four. (d) Plot of the principal axes two and three. (e) Plot of the principal axes two and four. (f) Plot of the principal axes three and four. The pie charts represent the proportions of membership established by fastSTRUCTURE for the best K = 5. **Supplementary Figure S6** Notched boxplot demonstrating the mean (white circle), the median (dark black line), 95% confidence interval around the median (notch), inter-quartile ranges (25 to 75%), whiskers (5 and 95%) and minimum and maximum measurements (crosses) of quantitative characters (a-q) for the studied species. Statistical significance was tested by Wilcoxon rank-sum test for post hoc group comparisons with Bonferroni correction, *p* < 0.001, *p* < 0.01, *p* < 0.05, *p* < 0.1 and *p* < 1 noted as ‘***’, ‘**’, ‘*’, ‘.’ and no symbol, respectively. Due to the small sample size, *p*-values cannot be properly estimated for *S. grandis* × *S. krylovii* and *S. grandis* × *S. baicalensis*. Each dot represents an observation. **Supplementary Figure S7** Bar charts displaying frequencies of the qualitative characters. (a) AbSVL. (b) AdSVL. (c) HTTA. (d) AG. (e) CN. (f) PHBN.

## Data Availability

The SNP dataset derived from the DArTseq pipeline in the genlight format is available via Figshare repository, 10.6084/m9.figshare.14461802.
