# Correction to: Evidence for extensive hybridization and past introgression events in feather grasses using genome-wide SNP genotyping

**DOI:** 10.1186/s12870-021-03357-z

**Published:** 2022-01-08

**Authors:** Evgenii Baiakhmetov, Daria Ryzhakova, Polina D. Gudkova, Marcin Nobis

**Affiliations:** 1 Institute of Botany, Faculty of Biology, Jagiellonian University, Gronostajowa 3, 30-387 Kraków, Poland; 2 Research laboratory ‘Herbarium’, National Research Tomsk State University, Lenin 36 Ave., Tomsk, 634050 Russia; 3 Department of Biology, Altai State University, Lenin 61 Ave, Barnaul, 656049 Russia

**Correction to: BMC Plant Biol 21, 505 (2021)**

**https://doi.org/10.1186/s12870-021-03287-w**

Following publication of the original article [1], the author identified an error in Supplementary Materials. Additional File 1, Interactive box plots, is missing. Figures 1, 2, 3, 4, 5, 6 and 7.

**Fig. 1:**
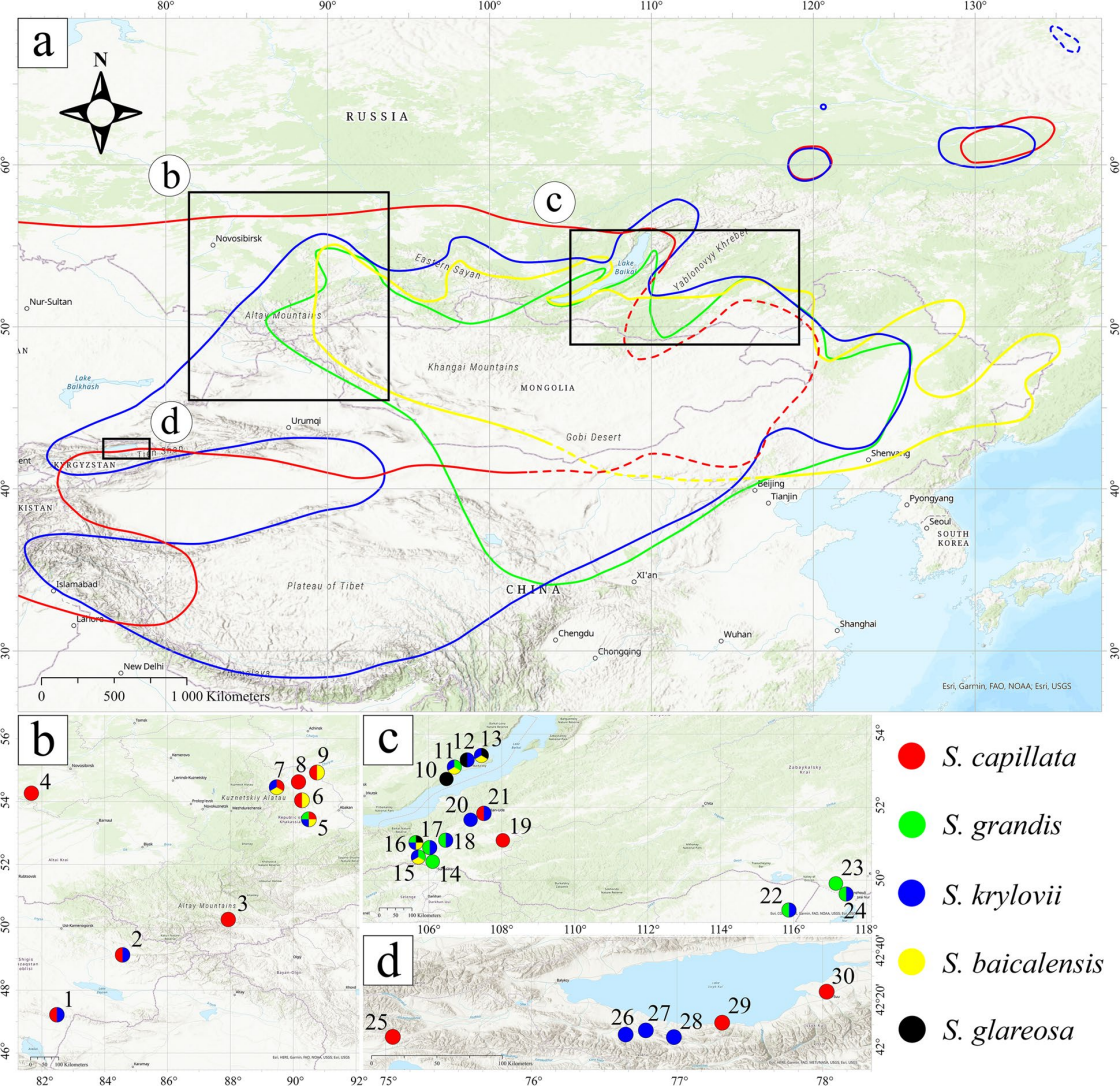
The general distribution map of (**a**) *S. baicalensis* (yellow), *S. capillata* (red), *S. grandis* (green), *S. krylovii* (blue) and sampling locations (**b**) in East Kazakhstan and southwestern Siberia (Russia), (**c**) in southeastern Siberia and (d) in Eastern Kyrgyzstan. The dashed lines indicate hypothetical borders. The coloured circles depict species found in the numbered locations. The exact coordinates of the locations are presented in the Supplementary Table S1

**Fig. 2:**
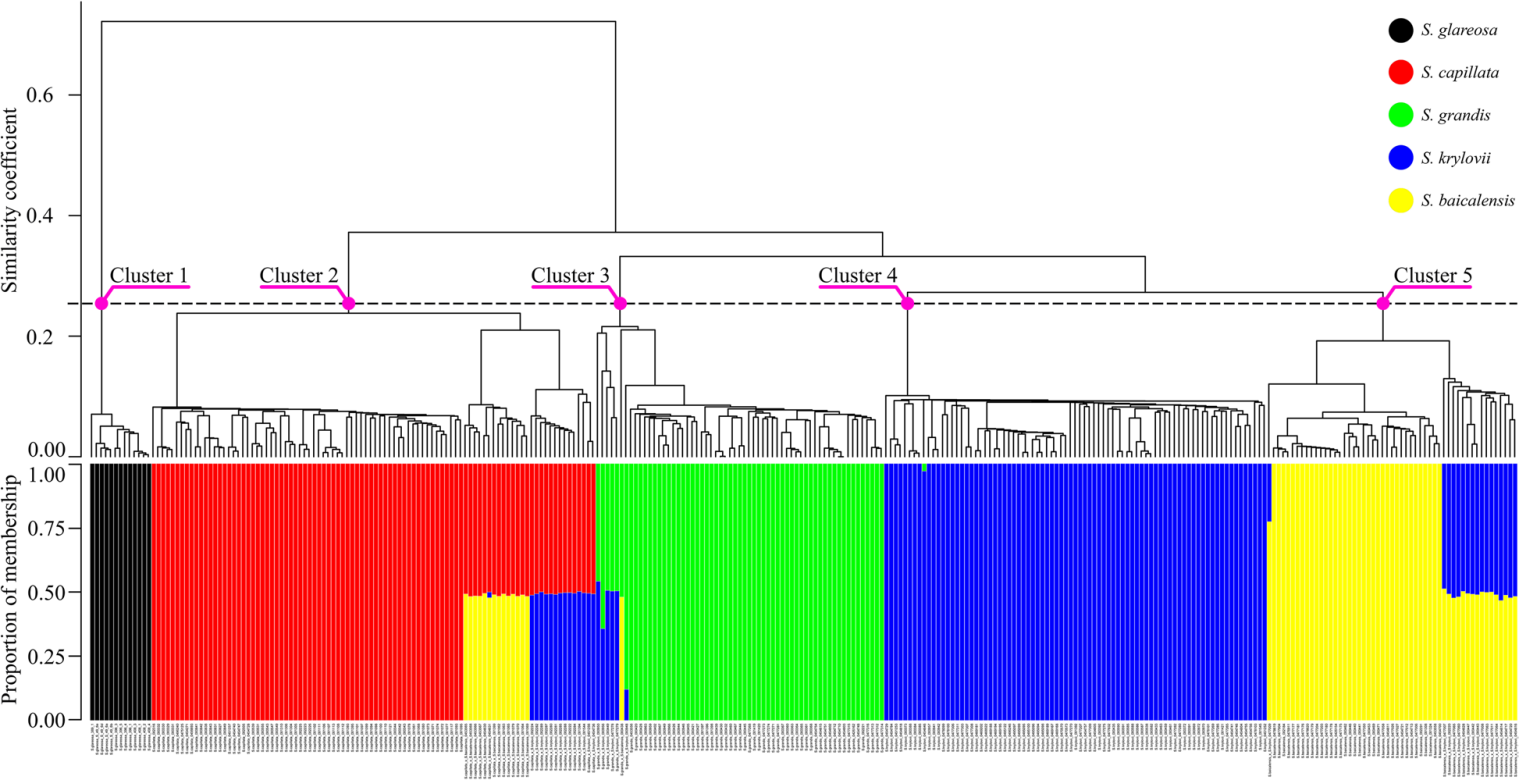
The UPGMA dendrogram (at the top) aligned with the best supported fastSTRUCTURE model K = 5 (on the bottom). The genetic distance was calculated using the Jaccard Similarity Coefficient (y-axis, top). Individuals are represented by coloured bars according to the proportion of membership (y-axis, bottom) of a genotype to the respective cluster

**Fig. 3:**
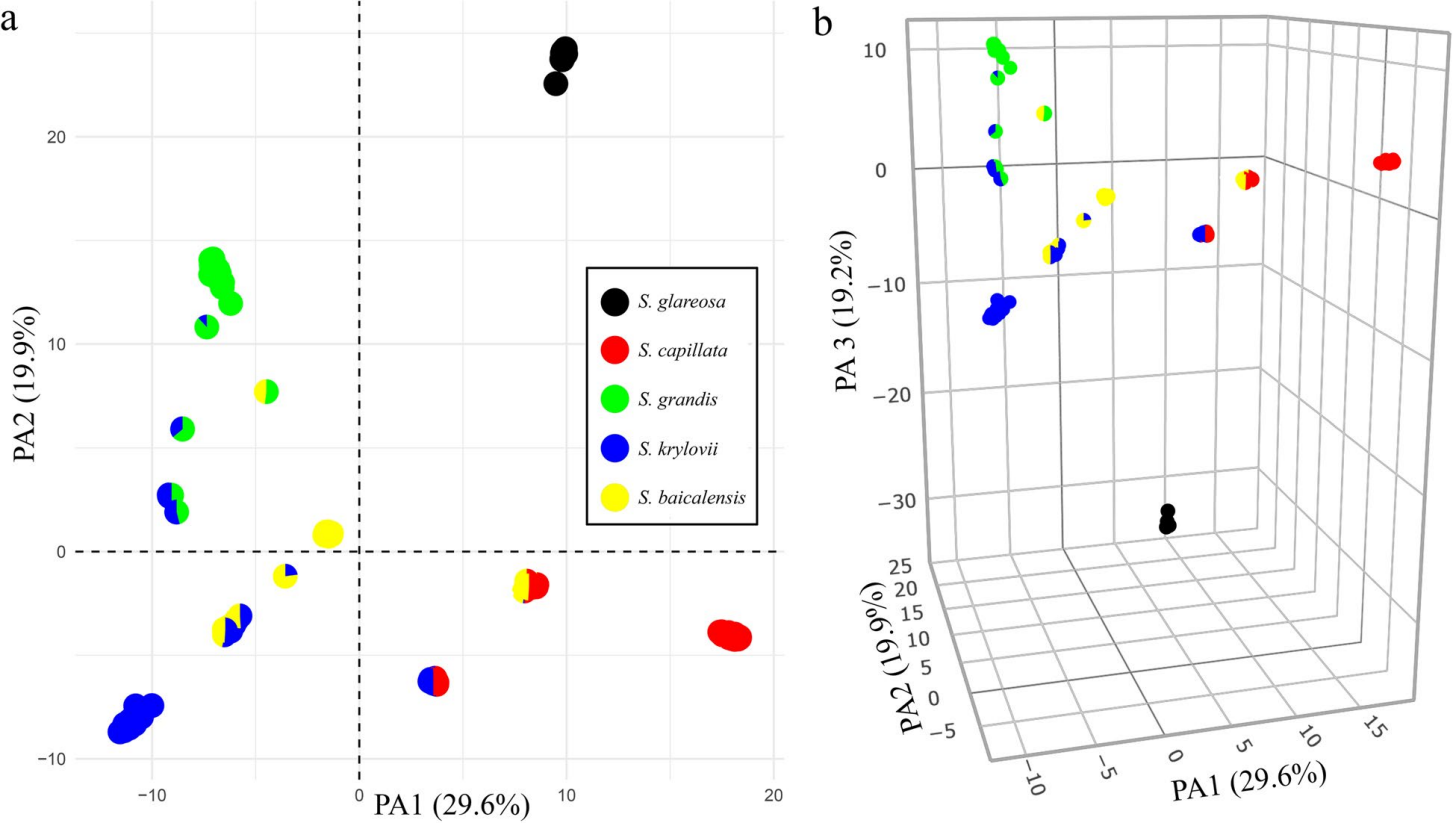
The PCoA plot based on genetic distances between samples. **a** The plot of the two principal axes. **b** The plot of the three principal axes. The pie charts represent the proportions of membership established by fastSTRUCTURE for the best K = 5

**Fig. 4:**
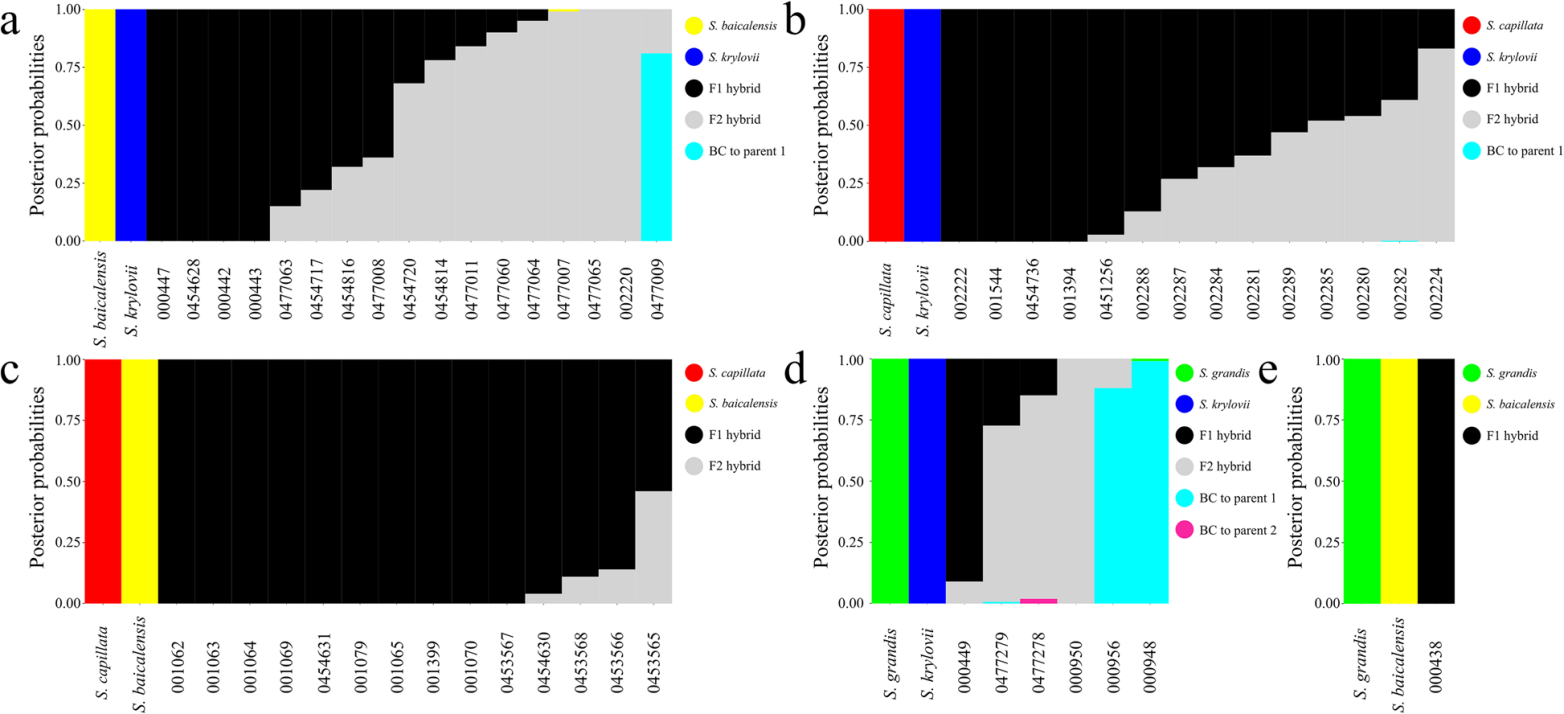
The assignment of *Stipa* taxa into four hybrid classes according to the posterior probabilities (y-axis) inferred in NewHybrids. **a** *S. baicalensis* × *S. krylovii*, (**b**) *S. capillata* × *S. krylovii*, (**c**) *S. capillata* × *S. baicalensis*, (**d**) *S. grandis* × *S. krylovii*, (**e**) *S. grandis* × *S. baicalensis*. Hybrid classes are coloured by black (F1 hybrid), grey (F2), cyan (backcross to the first parental species, BC to parent 1) and pink (backcross to the second parental species, BC to parent 2)

**Fig. 5:**
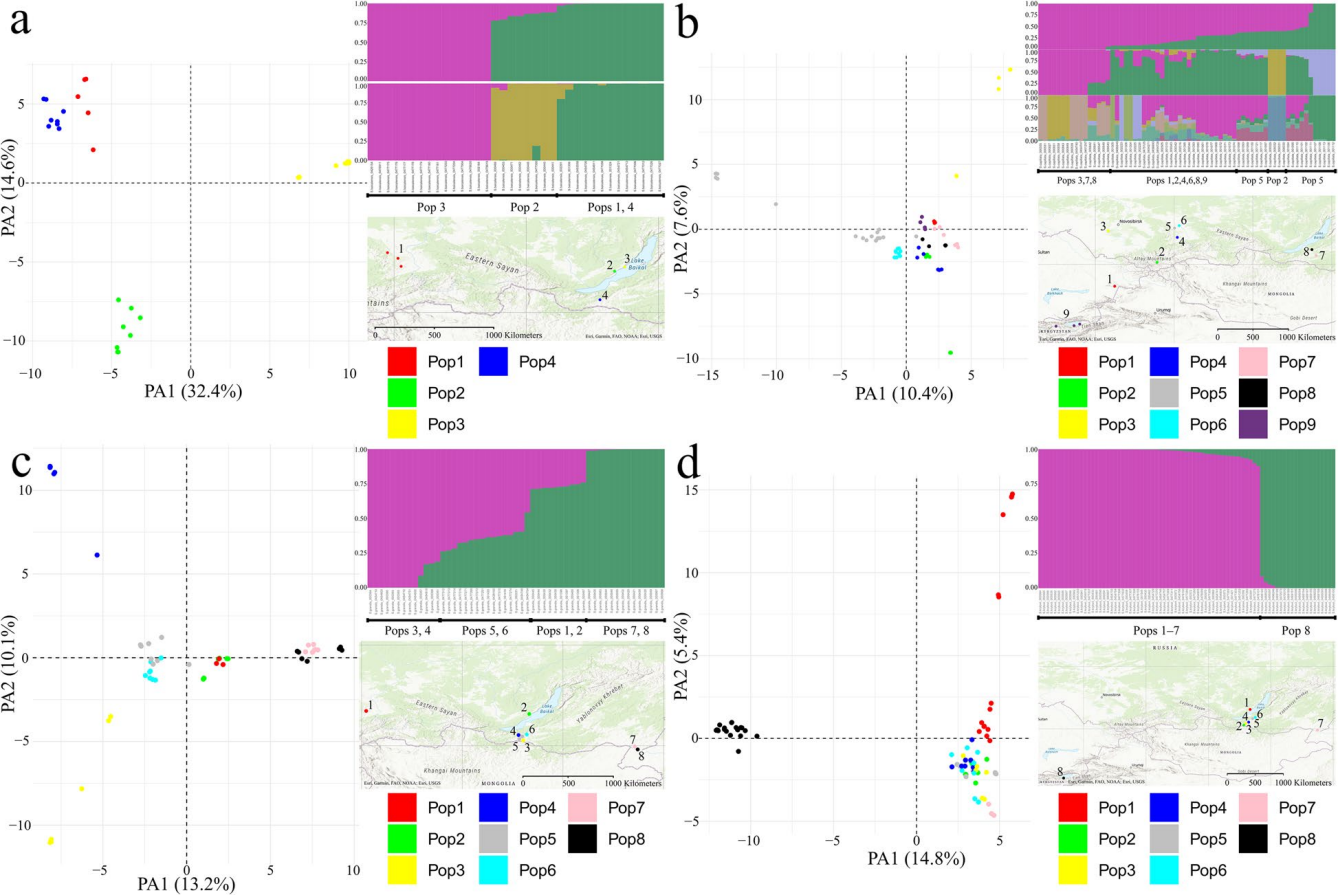
PCoA plots, best supported STRUCTURE models and localities of the studied populations across four species. **a** *S. baicalensis*. **b** *S. capillata.* **c** *S. grandis.* **d** *S. krylovii*

**Fig. 6:**
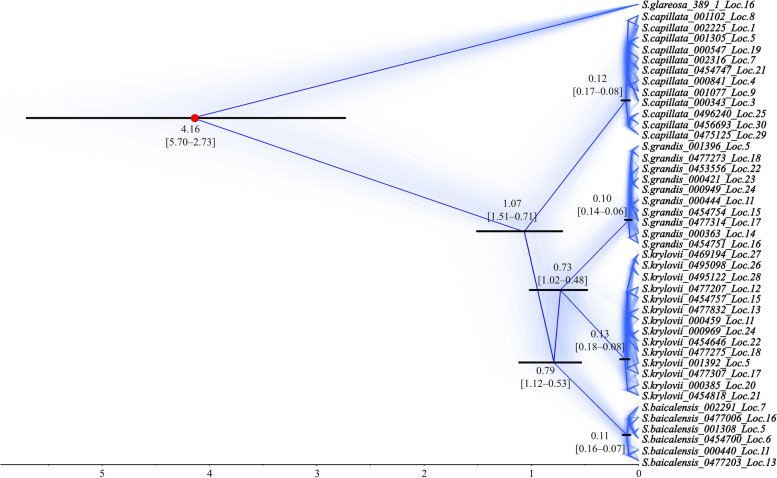
Phylogeny and divergence date estimates inferred by SNAPP. Blue coloured trees represent the most probable topology. Numbers at each node represent mean ages of divergence time estimates and the 95% HPD intervals (in the brackets). The black rectangles on the nodes indicate the 95% HPD intervals of the estimated posterior distributions of the divergence times. The red circle indicates the presumed divergence time split set as a reference. The Bayesian posterior probabilities were 1.00 for the nodes with the shown 95% HPD intervals. The scale shows divergence time in Mya

**Fig. 7:**
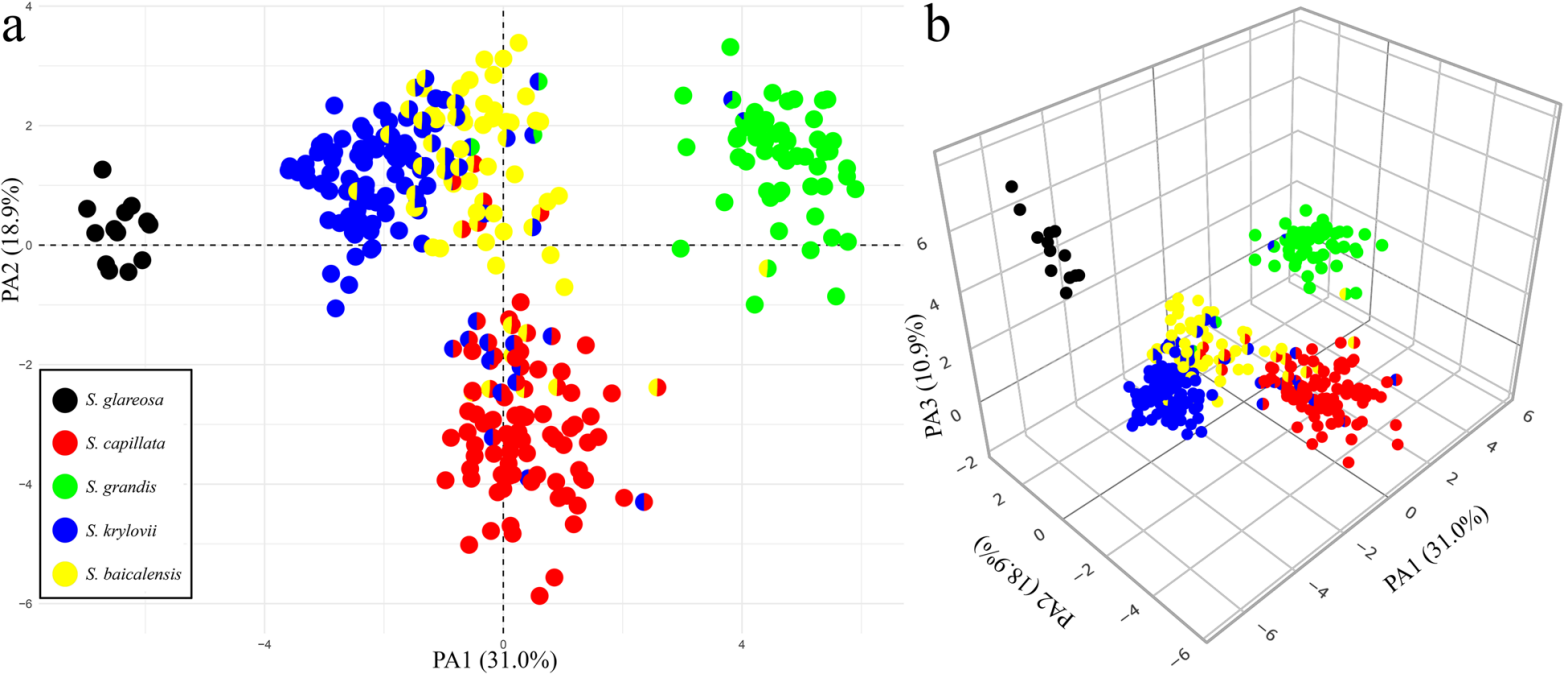
The factor analysis of mixed data performed on 17 quantitative and six qualitative characters of the five examined species of *Stipa*. **a** Plot of the two principal axes. **b** Plot of the three principal axes. The pie charts represent the proportions of membership established by fastSTRUCTURE for the best K = 5

Additionally, revised and high resolution figures should also be captured. The revised figures are given below:

The original article has been corrected.

## Supplementary Information

**Additional file 1.**
