# Supplementary material for: Evidence for extensive hybridisation and past introgression events in feather grasses using genome-wide SNP genotyping
Source: BMC Plant Biol. 2021 Nov 1;21:505. doi: 10.1186/s12870-021-03287-w (PMC8559405; doi:10.1186/s12870-021-03287-w)
Supplement: Supplementary file 2 — Additional file 2: Supplementary Table S1. List of samples used in the study. Table S2. Average posterior probabilities inferred in NewHybrids for first (F1) and second (F2) generation hybrids and backcrosses (F1xP1 and F1xP2). Table S3. Pairwise Fst values for population differentiation across the four studied species. Fst > 0.15 indicating high levels of differentiation are in bold type. Table S4. Contribution (%) by dimension of each character (abbreviations according to Table 3) in FAMD. The first five characters contributing the most are in bold type. Abbreviations of the qualitative variables and their contributions to the principal axes are underlined. Table S5. The assigned species names based on morphological and molecular data. Mismatches are shown in bold type. Supplementary Figure S1 Venn diagram representing polymorphic SNPs among four pure Stipa species. The admixed individuals and S. glareosa, which did not show patterns of hybridisation, were omitted in the metric’s calculation. Supplementary Figure S2 Delta K values calculated by Evanno’s method across four species. (a) S. baicalensis. (b) S. capillata. (c) S. grandis. (d) S. krylovii. Supplementary Figure S3 Phylogeny (at the top) and divergence date estimates at the species level (on the bottom) inferred by SNAPP. The scale shows divergence time in Mya. The red circles indicate nodes with the Bayesian posterior probabilities (BPP) > 0.8. The lower-case letters refer to the embedded table containing data regarding the exact estimates of the divergence times (in kya), BPPs and 95% HPD intervals. Supplementary Figure S4 Correlation matrix of the studied morphological characters (abbreviations according to Table 3). Colour intensity and the size of the circle are proportional to the correlation coefficients (displayed in the circle). Positive correlations are blue while negative are red. All p-values of Pearson correlations were < 0.01. Supplementary Figure S5 Factor analysis of mixed data performed o [file 12870_2021_3287_MOESM2_ESM.docx]

Evidence for extensive hybridisation and past introgression events in feather grasses using genome-wide SNP genotyping

Evgenii Baiakhmetov^1,2*^, Daria Ryzhakova^2,3^, Polina D. Gudkova^2,3^, Marcin Nobis^1,2*^

^1^ Institute of Botany, Faculty of Biology, Jagiellonian University, Gronostajowa 3, 30-387 Kraków, Poland

^2^ Research laboratory 'Herbarium', National Research Tomsk State University, Lenin 36 Ave., 634050 Tomsk, Russia

^3^ Department of Biology, Altai State University, Lenin 61 Ave., 656049 Barnaul, Russia

*Corresponding Authors:

Evgenii Baiakhmetov^1,2^

Gronostajowa 3, Kraków, 30-387 Kraków, Poland

Email address: evgenii.baiakhmetov@doctoral.uj.edu.pl

Marcin Nobis^1,2^

Gronostajowa 3, Kraków, 30-387 Kraków, Poland

Email address: m.nobis@uj.edu.pl

Supplementary Table S1. List of samples used in the study.

| Taxon  (assigned by genetics) | Voucher No in TK/KRA | Locality No, description | Latitude | Longitude | Altitude | Date | Collector |
| --- | --- | --- | --- | --- | --- | --- | --- |
| *S. capillata*  *S. capillata*  *S. capillata*  *S. capillata × S. krylovii*  *S. capillata × S. krylovii* | 002223  002225  002226  002222  002224 | Locality 1,  Kazakhstan,  Tarbagatai Mountains,  Khrebet Tarbagatay | N 47°11'17.9"  (approx.) | E 82°27'59.0"  (approx.) | 2978 m | 21.06.2005 | Y.A.Kotukhov |
| *S. capillata × S. krylovii*  *S. capillata × S. krylovii*  *S. capillata × S. krylovii*  *S. capillata × S. krylovii*  *S. capillata × S. krylovii*  *S. capillata × S. krylovii*  *S. capillata × S. krylovii*  *S. capillata × S. krylovii* | 002280  002281  002282  002284  002285  002287  002288  002289 | Locality 2,  Kazakhstan,  Katonkaragay District,  NW of Khrebet Sarymsakty | N 49°06'15.6"  (approx.) | E 84°33'30.8"  (approx.) | 1400 m | 18.08.2004 | Y.A.Kotukhov |
| *S. capillata*  *S. capillata*  *S. capillata*  *S. capillata*  *S. capillata*  *S. capillata*  *S. capillata* | 000329  000330  000331  000332  000342  000343  000344 | Locality 3,  Russia,  Altai Republic,  Kuray | N 50°13'58.3'' | E 87°55'48.7'' | 1495 m | 23.08.2018 | L.Sokolova |
| *S. capillata*  *S. capillata*  *S. capillata*  *S. capillata*  *S. capillata*  *S. capillata*  *S. capillata* | 000827  000831  000838  000841  000842  000843  000849 | Locality 4,  Russia,  Novosibirskaya oblast,  Kirza | N 54°15'32.1" | E 81°39'29.1" | 95 m | 27.09.2018 | E.Kriuchkova |
| *S. capillata*  *S. capillata*  *S. capillata*  *S. baicalensis*  *S. baicalensis* | 001304  001305  001306  001308  001324 | Locality 5,  Russia,  Republic of Khakassia,  Askizsky District,  NW shore of Lake Bulankul' | N 53°27'43.8" | E 90°24'03.8" | 844 m | 22.07.2008 | M.Olonova  P.Gudkova |
| *S. capillata* | 001310 | Locality 5,  Russia,  Republic of Khakassia,  Askizsky District,  ca. 11 km SE of Lake Bulankul' | N 53°25'37.7" | E 90°34'39.3" | 452 m | 24.07.2008 | P.Gudkova |
| *S. capillata*  *S. capillata*  *S. capillata × S. baicalensis*  *S. capillata × S. krylovii*  *S. grandis*  *S. grandis*  *S. grandis*  *S. krylovii* | 001393  001395  001399  001394  001396  001397  001398  001392 | Locality 5,  Russia,  Republic of Khakassia,  Askizsky District,  ca. 14 km NW of Kamyshta | N 53°21'32.9" | E 90°33'27.3" | 421 m | 23.07.2008 | P.Gudkova |
| *S. baicalensis*  *S. baicalensis*  *S. capillata × S. baicalensis*  *S. capillata × S. baicalensis*  *S. capillata × S. baicalensis*  *S. capillata × S. baicalensis* | 0453558  0454700  0453565  0453566  0453567  0453568 | Locality 6,  Russia,  Republic of Khakassia,  Shirinskiy District,  6 km N of Erbinskaya | N 54°02'05.0"  (approx.) | E 90°16'04.7"  (approx.) | 742 m | 21.07.2008 | P.Gudkova  M.Olonova |
| *S. baicalensis × S. krylovii* | 0454628 | Locality 7,  Russia,  Republic of Khakassia,  Shirinskiy District,  Yefremkino | N 54°28'18.0" | E 89°27'42.0" | 543 m | 08.08.2013 | A.Ebel |
| *S. capillata × S. baicalensis*  *S. capillata × S. baicalensis* | 0454630  0454631 | Locality 7,  Russia,  Republic of Khakassia,  Shirinskiy District,  ca. 1 km SE of Yefremkino | N 54°27'38.0" | E 89°28'15.0" | 592 m | 10.08.2013 | A.Ebel |
| *S. baicalensis* | 002291 | Locality 7,  Russia,  Republic of Khakassia,  Shirinskiy District,  ca. 1.5 km SE of Yefremkino | N 54°27'03.3" | E 89°27'35.9" | 552 m | 15.08.2013 | A.Ebel |
| *S. capillata* | 002316 | Locality 7,  Russia,  Republic of Khakassia,  Shirinskiy District,  ca. 5 km SE of Yefremkino | N 54°26'09.8" | E 89°30'59.8" | 892 m | 11.08.2013 | A.Ebel |
| *S. capillata*  *S. capillata*  *S. capillata*  *S. capillata*  *S. capillata*  *S. capillata*  *S. capillata*  *S. capillata*  *S. capillata*  *S. capillata*  *S. capillata*  *S. capillata*  *S. capillata*  *S. capillata*  *S. capillata*  *S. capillata*  *S. capillata*  *S. capillata* | 001093  001094  001095  001097  001098  001099  001100  001101  001102  001106  001107  001108  001110  001111  001112  001113  001117  001121 | Locality 8,  Russia,  Republic of Khakassia,  SE of Lake Bele | N 54°36'41'' | E 90°10'06.3'' | 435 m | 20.08.2018 | P.Gudkova |
| *S. capillata*  *S. capillata*  *S. capillata*  *S. capillata*  *S. capillata*  *S. capillata*  *S. capillata*  *S. capillata*  *S. capillata*  *S. capillata × S. baicalensis*  *S. capillata × S. baicalensis*  *S. capillata × S. baicalensis*  *S. capillata × S. baicalensis*  *S. capillata × S. baicalensis*  *S. capillata × S. baicalensis*  *S. capillata × S. baicalensis* | 001061  001068  001071  001072  001073  001074  001077  001078  001080  001062  001063  001064  001065  001069  001070  001079 | Locality 9,  Russia,  Krasnoyarsk Krai,  ca. 10.5 W of Yenisei River' bank | N 54°54'50.6'' | E 90°45'09.9'' | 493 m | 20.08.2018 | P.Gudkova |
| *S. glareosa*  *S. glareosa*  *S. glareosa*  *S. glareosa* | 0426149 3 \| 396_3  0426149 4 \| 396_4  0426149 5 \| 396_5  0426150 \| 396_1 | Locality 10,  Russia,  Irkutsk Oblast,  W shore of Lake Baikal | N 52°52'42.25" | E 106°35'7.77" | 597 m | 02.08.2014 | M.Nobis |
| *S. baicalensis* | 0477350 | Locality 11,  Russia,  Irkutsk Oblast,  W shore of Lake Baikal | N 53°1'23.52" | E 106°43'58.91" | 473 m | 04.08.2014 | M.Nobis |
| *S. baicalensis*  *S. baicalensis*  *S. baicalensis*  *S. baicalensis*  *S. baicalensis*  *S. baicalensis*  *S. baicalensis*  *S. baicalensis × S. krylovii*  *S. baicalensis × S. krylovii*  *S. baicalensis × S. krylovii*  *S. grandis*  *S. grandis*  *S. grandis*  *S. grandis*  *S. grandis*  *S. grandis*  *S. grandis*  *S. grandis × S. baicalensis*  *S. grandis × S. krylovii*  *S. krylovii*  *S. krylovii*  *S. krylovii*  *S. krylovii*  *S. krylovii* | 000440  000441  000446  000453  000462  000469  000471  000442  000443  000447  000430  000432  000439  000444  000445  000460  000467  000438  000449  000433  000435  000459  000461  000463 | Locality 11,  Russia,  Irkutsk Oblast,  W shore of Lake Baikal | N 53°02'58.3" | E 106°44'56.7" | 502 m | 18.08.2018 | P.Gudkova |
| *S. krylovii*  *S. krylovii*  *S. krylovii*  *S. krylovii*  *S. krylovii*  *S. krylovii*  *S. krylovii*  *S. krylovii* | 000538  000582  000585  000586  000587  000588  000590  000591 | Locality 11,  Russia,  Irkutsk Oblast,  W shore of Lake Baikal | N 53°05'18.0" | E 106°48'37.0" | 501 m | 18.08.2018 | P.Gudkova |
| *S. glareosa*  *S. glareosa*  *S. glareosa*  *S. glareosa* | 0477208 1 \| 408_1  0477208 2 \| 408_2  0477209 3 \| 408_3  0477209 4 \| 408_4 | Locality 12,  Russia,  Irkutsk Oblast,  W shore of Lake Baikal | N 53°9'10.58" | E 106°57'30.47" | 465 m | 03.08.2014 | M.Nobis |
| *S. krylovii* | 0477207 | Locality 12,  Russia,  Irkutsk Oblast,  W shore of Lake Baikal | N 53°10'47.50" | E 106°57'48.89" | 467 m | 03.08.2014 | M.Nobis |
| *S. baicalensis* | 0477904 | Locality 13,  Russia,  Irkutsk Oblast,  W shore of Lake Baikal | N 53°24'19.77" | E 107°26'21.61" | 470 m | 03.08.2014 | M.Nobis |
| *S. baicalensis*  *S. baicalensis*  *S. baicalensis*  *S. baicalensis*  *S. baicalensis*  *S. baicalensis*  *S. baicalensis* | 002184  0426154  0476911  0477203  0477204  0478619  0478620 | Locality 13,  Russia,  Irkutsk Oblast,  W shore of Lake Baikal | N 53°24'17.1" | E 107°26'18.5" | 450 m | 03.08.2014 | P.Gudkova  M.Nobis  A.Ebel |
| *S. glareosa*  *S. glareosa*  *S. glareosa*  *S. glareosa* | 0457716 A \| 49_6a  0457716 B \| 49_6b  0454146 \| 49_6d  0454147 \| 49_6e | Locality 13,  Russia,  Irkutsk Oblast,  W shore of Lake Baikal | N 53°24'14" | E 107°26'12" | 473 m | 21.07.2013 | A.Ebel |
| *S. baicalensis*  *S. baicalensis*  *S. baicalensis*  *S. baicalensis*  *S. baicalensis*  *S. baicalensis*  *S. baicalensis*  *S. krylovii* | 0477175  0477176  0477177  0477178  0477179  0477180  0477181  0477832 | Locality 13,  Russia,  Irkutsk Oblast,  W shore of Lake Baikal | N 53°24'19.79" | E 107°26'21.68" | 465 m | 03.08.2014 | M.Nobis |
| *S. grandis*  *S. grandis*  *S. grandis*  *S. grandis*  *S. grandis*  *S. grandis*  *S. grandis* | 000360  000361  000363  000364  000365  000367  000368 | Locality 14,  Russia,  Republic of Buryatia,  ca.2 km W of Dyrestuy | N 50°38'50.1" | E 106°01'45.1" | 628 m | 16.08.2018 | P.Gudkova |
| *S. baicalensis × S. krylovii*  *S. grandis*  *S. grandis*  *S. krylovii*  *S. krylovii*  *S. krylovii*  *S. krylovii*  *S. krylovii*  *S. krylovii*  *S. krylovii* | 002220  0454748  0454754  0454741  0454746  0454755  0454756  0454757  0454804  0454805 | Locality 15,  Russia,  Republic of Buryatia,  ca. 5 km NW of Borgoy | N 50°47'37.89" | E 105°47'12.01" | 780 m | 31.07.2014 | M.Nobis |
| *S. baicalensis*  *S. baicalensis*  *S. baicalensis*  *S. baicalensis*  *S. baicalensis*  *S. baicalensis*  *S. baicalensis*  *S. baicalensis*  *S. baicalensis × S. krylovii*  *S. baicalensis × S. krylovii*  *S. baicalensis × S. krylovii*  *S. baicalensis × S. krylovii*  *S. baicalensis × S. krylovii*  *S. baicalensis × S. krylovii*  *S. baicalensis × S. krylovii*  *S. baicalensis × S. krylovii*  *S. baicalensis × S. krylovii*  *S. baicalensis × S. krylovii*  *S. baicalensis × S. krylovii*  *S. baicalensis × S. krylovii*  *S. glareosa*  *S. grandis*  *S. grandis*  *S. grandis*  *S. grandis*  *S. grandis*  *S. grandis* | 0454721  0454713  0454811  0477006  0477012  0477026  0477027  0477058  0454717  0454720  0454814  0454816  0477007  0477008  0477009  0477011  0477060  0477063  0477064  0477065  0477252 \| 389_1  0454710  0454712  0454751  0454802  0454803  0454815 | Locality 16,  Russia,  Republic of Buryatia,  ca. 6 km NW of Inzagatuy | N 50°52'50.04" | E 105°41'48.62" | 1080 m | 1.08.2014 | M.Nobis |
| *S. grandis*  *S. grandis*  *S. grandis*  *S. grandis*  *S. grandis*  *S. grandis*  *S. krylovii*  *S. krylovii*  *S. krylovii*  *S. krylovii*  *S. krylovii*  *S. krylovii* | 0426162  0477312  0477313  0477314  0477315  0477316  0477303  0477306  0477307  0477308  0477309  0477311 | Locality 17,  Russia,  Republic of Buryatia,  ca. 14 km E of Inzagatuy | N 50°49'9.35" | E 105°56'43.63" | 870 m | 31.07.2014 | M.Nobis |
| *S. grandis*  *S. grandis*  *S. grandis*  *S. grandis*  *S. grandis*  *S. grandis*  *S. grandis*  *S. grandis*  *S. grandis × S. krylovii*  *S. grandis × S. krylovii*  *S. krylovii*  *S. krylovii*  *S. krylovii*  *S. krylovii*  *S. krylovii*  *S. krylovii*  *S. krylovii*  *S. krylovii* | 001419  001420  002221  0477268  0477271  0477273  0477274  0477281  0477278  0477279  001421  0477265  0477269  0477270  0477275  0477280  0477282  0477285 | Locality 18,  Russia,  Republic of Buryatia,  ca. 7 km SE of Lake Gusinoye | N 51°05'6.17" | E 106°28'24.56" | 778 m | 31.07.2014 | P.Gudkova  M.Nobis  A.Ebel |
| *S. capillata*  *S. capillata*  *S. capillata*  *S. capillata*  *S. capillata*  *S. capillata* | 000531  000539  000543  000547  000549  000555 | Locality 19,  Russia,  Republic of Buryatia,  16 km W of Khonkholoy | N 51°06'02.0" | E 108°01'57.0" | 812 m | 15.08.2018 | P.Gudkova |
| *S. krylovii*  *S. krylovii*  *S. krylovii*  *S. krylovii*  *S. krylovii*  *S. krylovii*  *S. krylovii* | 000372  000373  000384  000385  000388  000391  000392 | Locality 20,  Russia,  Republic of Buryatia,  ca. 14 km NE of Orongoy | N 51°38'07.7'' | E 107°08'51.2'' | 691 m | 17.08.2018 | P.Gudkova |
| *S. capillata*  *S. capillata*  *S. capillata*  *S. capillata*  *S. capillata*  *S. capillata × S. krylovii*  *S. capillata × S. krylovii*  *S. capillata × S. krylovii*  *S. krylovii*  *S. krylovii*  *S. krylovii*  *S. krylovii*  *S. krylovii*  *S. krylovii*  *S. krylovii*  *S. krylovii*  *S. krylovii*  *S. krylovii* | 0451257  0454728  0454740  0454747  002505  0451256  0454736  001544  001545  0451252  0451253  0454729  0454784  0454818  0478099  0478100  0478101  0478103 | Locality 21,  Russia,  Republic of Buryatia,  main road Ulan-Ude - Zarechny | N 51°49'34.90" | E 107°30'23.02'' | 495 m | 30.07.2014 | P.Gudkova  M.Nobis  A.Ebel |
| *S. grandis*  *S. krylovii* | 0453556  0454646 | Locality 22,  Mongolia,  Khovd Province,  ca. 27 km SW of Khar Lake | N 49°10'47.9" | E 115°51'55.1" | 713 m | 12.08.2011 | I.N.Safronova  N.Narantuya  T.Uchrakhbayar |
| *S. grandis*  *S. grandis*  *S. grandis*  *S. grandis*  *S. grandis*  *S. grandis*  *S. grandis* | 000402  000405  000420  000421  000426  000427  000428 | Locality 23,  Russia,  Zabaykalsky Krai,  ca. 6 km E of Bilyutuj | N 49°48'40.0" | E 117°09'54.0" | 786 m | 13.08.2018 | P.Gudkova |
| *S. grandis*  *S. grandis*  *S. grandis*  *S. grandis*  *S. grandis*  *S. grandis*  *S. grandis*  *S. grandis × S. krylovii*  *S. grandis × S. krylovii*  *S. grandis × S. krylovii*  *S. krylovii*  *S. krylovii*  *S. krylovii*  *S. krylovii*  *S. krylovii*  *S. krylovii* | 000944  000946  000949  000959  000963  000966  000967  000948  000950  000956  000941  000942  000957  000969  000970  000972 | Locality 24,  Russia,  Zabaykalsky Krai,  Zabaikal'sk | N 49°39'39.6'' | E 117°21'30.4'' | 729 m | 13.08.2018 | P.Gudkova |
| *S. capillata* | 0496240 | Locality 25,  Kyrgyzstan,  Central Tian Shan,  ca. 47 km NNE of Chaek | N 42°4'41.29" | E 75°3'12.43" | 2387 m | 07.07.2018 | M.Nobis  E.Klichowska  A.Wróbel  A.Nowak |
| *S. krylovii*  *S. krylovii*  *S. krylovii*  *S. krylovii*  *S. krylovii*  *S. krylovii*  *S. krylovii* | 0495094  0495095  0495096  0495097  0495098  0495099  0495100 | Locality 26,  Kyrgyzstan,  SW of Lake Issyk-Kul,  3 km E of Kongurlen | N 42°5'47.07'' | E 76°39'6.22'' | 1940 m | 06.07.2017 | M.Nobis  E.Klichowska  A.Wróbel  A.Nowak |
| *S. krylovii* | 0468522 | Locality 27,  Kyrgyzstan,  SW of Lake Issyk-Kul,  3 km E of Kongurlen | N 42°6'2.04'' | E 76°46'35.41'' | 2040 m | 10.07.2015 | M.Nobis  A.Nowak |
| *S. krylovii*  *S. krylovii* | 0470570  0470573 | Locality 27,  Kyrgyzstan,  SW of Lake Issyk-Kul,  5 km of Kongurlen | N 42°6'36.66'' | E 76°47'18.16'' | 2040 m | 10.07.2015 | M.Nobis  A.Nowak |
| *S. krylovii*  *S. krylovii* | 0469167  0469168 | Locality 27,  Kyrgyzstan,  SW part of Lake Issyk-Kul,  15 km W of Bokonbayevo | N 42°8'10.30'' | E 76°48'11.84'' | 1900 m | 01.08.2016 | M.Nobis  A.Nobis |
| *S. krylovii* | 0496246 | Locality 27,  Kyrgyzstan,  SW part of Lake Issyk-Kul,  ca. 15.5 km W of Bokonbayevo | N 42°8'15.86" | E 76°48'18.82" | 1894 m | 04.07.2018 | M.Nobis  E.Klichowska  A.Wróbel  A.Nowak |
| *S. krylovii*  *S. krylovii*  *S. krylovii*  *S. krylovii*  *S. krylovii*  *S. krylovii* | 0469181  0469188  0469189  0469194  0469195  0469202 | Locality 27,  Kyrgyzstan,  SW part of Lake Issyk-Kul,  20 km W of Bokonbayevo | N 42°8'26.50'' | E 76°45'25.55'' | 2030 m | 1.08.2016 | M.Nobis  A.Nobis |
| *S. krylovii* | 0495122 | Locality 28,  Kyrgyzstan,  SW of Lake Issyk-Kul,  4 km SSW of Bokonbayevo | N 42°4'46.02'' | E 76°58'39.87'' | 1946 m | 03.07.2018 | M.Nobis  E.Klichowska  A.Wróbel  A.Nowak |
| *S. capillata* | 0475125 | Locality 29,  Kyrgyzstan,  S part of Lake Issyk-Kul,  ca. 20 km W of Barskoon | N 42°10'43.78'' | E 77°18'24.06'' | 1612 m | 01.08.2016 | M.Nobis  A.Nobis |
| *S. capillata*  *S. capillata* | 0454271  0456693 | Locality 30,  Kyrgyzstan,  E part of Lake Issyk-Kul,  ca. 27 km SWW of Karakol | N 42°23'16.08'' | E 78°1'42.76'' | 1800 m | 17.06.2013 | M.Nobis  A.Nowak |

Table S2. Average posterior probabilities inferred in NewHybrids for first (F1) and second (F2) generation hybrids and backcrosses (F1xP1 and F1xP2).

| ID | Parent 1 (P1) | Parent 2 (P2) | F1 | F2 | F1xP1 | F1xP2 |
| --- | --- | --- | --- | --- | --- | --- |
| 000447 | *S. baicalensis* (0.00) | *S. krylovii* (0.00) | 1.00 | 0.00 | 0.00 | 0.00 |
| 0454628 | *S. baicalensis* (0.00) | *S. krylovii* (0.00) | 1.00 | 0.00 | 0.00 | 0.00 |
| 000442 | *S. baicalensis* (0.00) | *S. krylovii* (0.00) | 1.00 | 0.00 | 0.00 | 0.00 |
| 000443 | *S. baicalensis* (0.00) | *S. krylovii* (0.00) | 1.00 | 0.00 | 0.00 | 0.00 |
| 0477063 | *S. baicalensis* (0.00) | *S. krylovii* (0.00) | 0.85 | 0.15 | 0.00 | 0.00 |
| 0454717 | *S. baicalensis* (0.00) | *S. krylovii* (0.00) | 0.78 | 0.22 | 0.00 | 0.00 |
| 0454816 | *S. baicalensis* (0.00) | *S. krylovii* (0.00) | 0.68 | 0.32 | 0.00 | 0.00 |
| 0477008 | *S. baicalensis* (0.00) | *S. krylovii* (0.00) | 0.64 | 0.36 | 0.00 | 0.00 |
| 0454720 | *S. baicalensis* (0.00) | *S. krylovii* (0.00) | 0.32 | 0.68 | 0.00 | 0.00 |
| 0454814 | *S. baicalensis* (0.00) | *S. krylovii* (0.00) | 0.22 | 0.78 | 0.00 | 0.00 |
| 0477011 | *S. baicalensis* (0.00) | *S. krylovii* (0.00) | 0.16 | 0.84 | 0.00 | 0.00 |
| 0477060 | *S. baicalensis* (0.00) | *S. krylovii* (0.00) | 0.1 | 0.9 | 0.00 | 0.00 |
| 0477064 | *S. baicalensis* (0.00) | *S. krylovii* (0.00) | 0.05 | 0.95 | 0.00 | 0.00 |
| 0477007 | *S. baicalensis* (0.01) | *S. krylovii* (0.00) | 0.00 | 0.99 | 0.00 | 0.00 |
| 0477065 | *S. baicalensis* (0.00) | *S. krylovii* (0.00) | 0.00 | 1.00 | 0.00 | 0.00 |
| 002220 | *S. baicalensis* (0.00) | *S. krylovii* (0.00) | 0.00 | 1.00 | 0.00 | 0.00 |
| 0477009 | *S. baicalensis* (0.00) | *S. krylovii* (0.00) | 0.00 | 0.19 | 0.81 | 0.00 |
| 001062 | *S. capillata* (0.00) | *S. baicalensis* (0.00) | 1.00 | 0.00 | 0.00 | 0.00 |
| 001063 | *S. capillata* (0.00) | *S. baicalensis* (0.00) | 1.00 | 0.00 | 0.00 | 0.00 |
| 001064 | *S. capillata* (0.00) | *S. baicalensis* (0.00) | 1.00 | 0.00 | 0.00 | 0.00 |
| 001069 | *S. capillata* (0.00) | *S. baicalensis* (0.00) | 1.00 | 0.00 | 0.00 | 0.00 |
| 0454631 | *S. capillata* (0.00) | *S. baicalensis* (0.00) | 1.00 | 0.00 | 0.00 | 0.00 |
| 001079 | *S. capillata* (0.00) | *S. baicalensis* (0.00) | 1.00 | 0.00 | 0.00 | 0.00 |
| 001065 | *S. capillata* (0.00) | *S. baicalensis* (0.00) | 1.00 | 0.00 | 0.00 | 0.00 |
| 001399 | *S. capillata* (0.00) | *S. baicalensis* (0.00) | 1.00 | 0.00 | 0.00 | 0.00 |
| 001070 | *S. capillata* (0.00) | *S. baicalensis* (0.00) | 1.00 | 0.00 | 0.00 | 0.00 |
| 0453567 | *S. capillata* (0.00) | *S. baicalensis* (0.00) | 1.00 | 0.00 | 0.00 | 0.00 |
| 0454630 | *S. capillata* (0.00) | *S. baicalensis* (0.00) | 0.96 | 0.04 | 0.00 | 0.00 |
| 0453568 | *S. capillata* (0.00) | *S. baicalensis* (0.00) | 0.89 | 0.11 | 0.00 | 0.00 |
| 0453566 | *S. capillata* (0.00) | *S. baicalensis* (0.00) | 0.86 | 0.14 | 0.00 | 0.00 |
| 0453565 | *S. capillata* (0.00) | *S. baicalensis* (0.00) | 0.54 | 0.46 | 0.00 | 0.00 |
| 002222 | *S. capillata* (0.00) | *S. krylovii* (0.00) | 1.00 | 0.00 | 0.00 | 0.00 |
| 001544 | *S. capillata* (0.00) | *S. krylovii* (0.00) | 1.00 | 0.00 | 0.00 | 0.00 |
| 0454736 | *S. capillata* (0.00) | *S. krylovii* (0.00) | 1.00 | 0.00 | 0.00 | 0.00 |
| 001394 | *S. capillata* (0.00) | *S. krylovii* (0.00) | 1.00 | 0.00 | 0.00 | 0.00 |
| 0451256 | *S. capillata* (0.00) | *S. krylovii* (0.00) | 0.97 | 0.03 | 0.00 | 0.00 |
| 002288 | *S. capillata* (0.00) | *S. krylovii* (0.00) | 0.87 | 0.13 | 0.00 | 0.00 |
| 002287 | *S. capillata* (0.00) | *S. krylovii* (0.00) | 0.73 | 0.27 | 0.00 | 0.00 |
| 002284 | *S. capillata* (0.00) | *S. krylovii* (0.00) | 0.68 | 0.32 | 0.00 | 0.00 |
| 002281 | *S. capillata* (0.00) | *S. krylovii* (0.00) | 0.63 | 0.37 | 0.00 | 0.00 |
| 002289 | *S. capillata* (0.00) | *S. krylovii* (0.00) | 0.53 | 0.47 | 0.00 | 0.00 |
| 002285 | *S. capillata* (0.00) | *S. krylovii* (0.00) | 0.48 | 0.52 | 0.00 | 0.00 |
| 002280 | *S. capillata* (0.00) | *S. krylovii* (0.00) | 0.46 | 0.54 | 0.00 | 0.00 |
| 002282 | *S. capillata* (0.00) | *S. krylovii* (0.00) | 0.39 | 0.61 | 0.00 | 0.00 |
| 002224 | *S. capillata* (0.00) | *S. krylovii* (0.00) | 0.17 | 0.83 | 0.00 | 0.00 |
| 000449 | *S. grandis* (0.00) | *S. krylovii* (0.00) | 0.91 | 0.09 | 0.00 | 0.00 |
| 0477279 | *S. grandis* (0.00) | *S. krylovii* (0.00) | 0.27 | 0.72 | 0.01 | 0.00 |
| 0477278 | *S. grandis* (0.00) | *S. krylovii* (0.00) | 0.15 | 0.83 | 0.00 | 0.02 |
| 000950 | *S. grandis* (0.00) | *S. krylovii* (0.00) | 0.00 | 1.00 | 0.00 | 0.00 |
| 000956 | *S. grandis* (0.00) | *S. krylovii* (0.00) | 0.00 | 0.12 | 0.88 | 0.00 |
| 000948 | *S. grandis* (0.01) | *S. krylovii* (0.00) | 0.00 | 0.00 | 0.99 | 0.00 |
| 000438 | *S. grandis* (0.00) | *S. baicalensis* (0.00) | 1.00 | 0.00 | 0.00 | 0.00 |

Table S3. Pairwise *F*st values for population differentiation across the four studied species. *F*st > 0.15 indicating high levels of differentiation are in bold type.

| Species and population No | Pop1 | Pop2 | Pop3 | Pop4 | Pop5 | Pop6 | Pop7 | Pop8 |
| --- | --- | --- | --- | --- | --- | --- | --- | --- |
| *S. baicalensis*, Pop2 | **0.4135** |  |  |  |  |  |  |  |
| *S. baicalensis*, Pop3 | **0.6369** | **0.5759** |  |  |  |  |  |  |
| *S. baicalensis*, Pop4 | **0.3181** | **0.3981** | **0.6338** |  |  |  |  |  |
| *S. capillata*, Pop2 | **0.4829** |  |  |  |  |  |  |  |
| *S. capillata*, Pop3 | **0.5094** | **0.4180** |  |  |  |  |  |  |
| *S. capillata*, Pop4 | **0.4101** | **0.2803** | **0.3586** |  |  |  |  |  |
| *S. capillata*, Pop5 | **0.2995** | **0.2361** | **0.3001** | **0.1826** |  |  |  |  |
| *S. capillata*, Pop6 | **0.4086** | **0.2793** | **0.3562** | **0.2023** | 0.1280 |  |  |  |
| *S. capillata*, Pop7 | **0.5108** | **0.3941** | **0.4243** | **0.3045** | **0.2633** | **0.3191** |  |  |
| *S. capillata*, Pop8 | **0.5037** | **0.3750** | **0.4228** | **0.3141** | **0.2505** | **0.3051** | **0.3858** |  |
| *S. capillata*, Pop9 | **0.4806** | **0.3379** | **0.3700** | **0.2554** | **0.2031** | **0.2569** | **0.3542** | **0.3483** |
| *S. grandis*, Pop2 | **0.4212** |  |  |  |  |  |  |  |
| *S. grandis*, Pop3 | **0.5169** | **0.4544** |  |  |  |  |  |  |
| *S. grandis*, Pop4 | **0.6817** | **0.5633** | **0.6067** |  |  |  |  |  |
| *S. grandis*, Pop5 | **0.3033** | **0.2530** | **0.3185** | **0.4092** |  |  |  |  |
| *S. grandis*, Pop6 | **0.2674** | **0.2477** | **0.2669** | **0.3916** | 0.1037 |  |  |  |
| *S. grandis*, Pop7 | **0.3308** | **0.3094** | **0.4260** | **0.5139** | **0.2487** | **0.23586** |  |  |
| *S. grandis*, Pop8 | **0.3709** | **0.3425** | **0.4484** | **0.5488** | **0.2760** | **0.2536** | 0.0826 |  |
| *S. krylovii*, Pop2 | 0.1245 |  |  |  |  |  |  |  |
| *S. krylovii*, Pop3 | 0.1476 | 0.0853 |  |  |  |  |  |  |
| *S. krylovii*, Pop4 | 0.1039 | 0.0565 | 0.0917 |  |  |  |  |  |
| *S. krylovii*, Pop5 | **0.1608** | 0.1215 | **0.1539** | 0.1039 |  |  |  |  |
| *S. krylovii*, Pop6 | 0.1176 | 0.0753 | 0.1071 | 0.0583 | 0.1172 |  |  |  |
| *S. krylovii*, Pop7 | **0.1753** | 0.1440 | **0.1632** | 0.1291 | **0.1867** | 0.1333 |  |  |
| *S. krylovii*, Pop8 | **0.3998** | **0.4065** | **0.4405** | **0.3772** | **0.4330** | **0.3717** | **0.4589** |  |

All *F*st *p*-values estimated using 1,000 bootstrap replicates were significant (<0.05).

Table S4. Contribution (%) by dimension of each character (abbreviations according to Table 3) in FAMD. The first five characters contributing the most are in bold type. Abbreviations of the qualitative variables and their contributions to the principal axes are underlined.

| Character | Dimension 1 | Dimension 2 | Dimension 3 | Dimension 4 |
| --- | --- | --- | --- | --- |
| Col1L | **10.58619639** | 0.07367645 | 0.202549383 | 0.18640246 |
| DDL | **10.32673209** | 0.13019599 | 2.560111433 | 0.183466 |
| AL | **9.811198066** | 1.5076406 | 0.124465739 | 1.07464193 |
| Col2L | **8.702219729** | 3.15270516 | 2.323643064 | 0.15100557 |
| CL | **8.42450895** | 1.28938092 | 0.932046305 | 0.95617566 |
| CN | 7.728393001 | 2.09548241 | 3.400721764 | 3.20582721 |
| LG | 6.48681185 | 0.64031939 | 5.19796294 | 1.30962683 |
| DVL | 5.893750308 | 0.71262269 | 0.000433553 | **6.41863223** |
| AdSVL | 5.728157162 | 3.66953586 | 0.693651975 | **6.27220701** |
| SL | 4.91977364 | 6.13121788 | 0.071357255 | 5.81027345 |
| LHTA | 3.273728594 | **8.60372028** | 3.864392941 | 0.09398638 |
| PHBN | 3.260074501 | 3.42809654 | **9.401227363** | 6.22797984 |
| WVS | 3.189179462 | 4.8203688 | 0.178227035 | 0.02115708 |
| HLCol1 | 3.169875893 | 0.12185596 | **23.728834** | 0.71345245 |
| AG | 3.087430743 | 0.0110623 | **26.33751993** | 0.08913552 |
| LigC | 1.868036208 | **11.29939534** | 0.28022867 | 0.01064054 |
| HTTA | 1.814224832 | **11.95190258** | 0.152983336 | 2.23887609 |
| LigIV | 0.695227504 | **13.68239344** | 0.024167264 | 2.21552371 |
| CBL | 0.55201756 | 2.85797371 | **6.619372557** | 4.77825171 |
| AbSVL | 0.308017226 | **13.32355635** | 3.670205699 | 0.75242778 |
| LHV | 0.146868843 | 5.7926466 | 0.354540743 | **19.53995161** |
| HLCol2 | 0.020173036 | 0.95448297 | **9.402285559** | **19.97205261** |
| LHD | 0.007404409 | 3.74976776 | 0.479071495 | **17.77830633** |

Table S5. The assigned species names based on morphological and molecular data. Mismatches are shown in bold type.

| Herbarium ID number | Species name assigned by morphology | Species name assigned by molecular data |
| --- | --- | --- |
| 001304 | ***S. baicalensis*** | ***S. capillata*** |
| 001305 | ***S. baicalensis*** | ***S. capillata*** |
| 001101 | ***S. baicalensis*** | ***S. capillata*** |
| 001113 | ***S. baicalensis*** | ***S. capillata*** |
| 001121 | ***S. baicalensis*** | ***S. capillata*** |
| 001061 | ***S. baicalensis*** | ***S. capillata*** |
| 001073 | ***S. baicalensis*** | ***S. capillata*** |
| 002225 | ***S. baicalensis*** | ***S. capillata*** |
| 002226 | ***S. baicalensis*** | ***S. capillata*** |
| 0453565 | ***S. baicalensis*** | ***S. capillata × S. baicalensis*** |
| 0453566 | ***S. baicalensis*** | ***S. capillata × S. baicalensis*** |
| 0453567 | ***S. baicalensis*** | ***S. capillata × S. baicalensis*** |
| 0453568 | ***S. baicalensis*** | ***S. capillata × S. baicalensis*** |
| 0454630 | ***S. baicalensis*** | ***S. capillata × S. baicalensis*** |
| 0454631 | ***S. baicalensis*** | ***S. capillata × S. baicalensis*** |
| 001062 | ***S. baicalensis*** | ***S. capillata × S. baicalensis*** |
| 001063 | ***S. baicalensis*** | ***S. capillata × S. baicalensis*** |
| 001064 | ***S. baicalensis*** | ***S. capillata × S. baicalensis*** |
| 001069 | ***S. baicalensis*** | ***S. capillata × S. baicalensis*** |
| 000447 | ***S. baicalensis*** | ***S. baicalensis × S. krylovii*** |
| 0454717 | ***S. baicalensis*** | ***S. baicalensis × S. krylovii*** |
| 0454814 | ***S. baicalensis*** | ***S. baicalensis × S. krylovii*** |
| 0454816 | ***S. baicalensis*** | ***S. baicalensis × S. krylovii*** |
| 0477009 | ***S. baicalensis*** | ***S. baicalensis × S. krylovii*** |
| 0477063 | ***S. baicalensis*** | ***S. baicalensis × S. krylovii*** |
| 0477064 | ***S. baicalensis*** | ***S. baicalensis × S. krylovii*** |
| 0477065 | ***S. baicalensis*** | ***S. baicalensis × S. krylovii*** |
| 002222 | ***S. baicalensis*** | ***S. capillata × S. krylovii*** |
| 002224 | ***S. baicalensis*** | ***S. capillata × S. krylovii*** |
| 002280 | ***S. baicalensis*** | ***S. capillata × S. krylovii*** |
| 002281 | ***S. baicalensis*** | ***S. capillata × S. krylovii*** |
| 002282 | ***S. baicalensis*** | ***S. capillata × S. krylovii*** |
| 002284 | ***S. baicalensis*** | ***S. capillata × S. krylovii*** |
| 002285 | ***S. baicalensis*** | ***S. capillata × S. krylovii*** |
| 002287 | ***S. baicalensis*** | ***S. capillata × S. krylovii*** |
| 002288 | ***S. baicalensis*** | ***S. capillata × S. krylovii*** |
| 002289 | ***S. baicalensis*** | ***S. capillata × S. krylovii*** |
| 0453558 | *S. baicalensis* | *S. baicalensis* |
| 0454700 | *S. baicalensis* | *S. baicalensis* |
| 002291 | *S. baicalensis* | *S. baicalensis* |
| 0477904 | *S. baicalensis* | *S. baicalensis* |
| 0477350 | *S. baicalensis* | *S. baicalensis* |
| 0426154 | *S. baicalensis* | *S. baicalensis* |
| 0476911 | *S. baicalensis* | *S. baicalensis* |
| 0477203 | *S. baicalensis* | *S. baicalensis* |
| 0477204 | *S. baicalensis* | *S. baicalensis* |
| 0478619 | *S. baicalensis* | *S. baicalensis* |
| 0478620 | *S. baicalensis* | *S. baicalensis* |
| 0477175 | *S. baicalensis* | *S. baicalensis* |
| 0477176 | *S. baicalensis* | *S. baicalensis* |
| 0477177 | *S. baicalensis* | *S. baicalensis* |
| 0477178 | *S. baicalensis* | *S. baicalensis* |
| 0477179 | *S. baicalensis* | *S. baicalensis* |
| 0477180 | *S. baicalensis* | *S. baicalensis* |
| 0477181 | *S. baicalensis* | *S. baicalensis* |
| 000446 | *S. baicalensis* | *S. baicalensis* |
| 000453 | *S. baicalensis* | *S. baicalensis* |
| 000462 | *S. baicalensis* | *S. baicalensis* |
| 000469 | *S. baicalensis* | *S. baicalensis* |
| 000471 | *S. baicalensis* | *S. baicalensis* |
| 0454721 | *S. baicalensis* | *S. baicalensis* |
| 0454713 | *S. baicalensis* | *S. baicalensis* |
| 0454811 | *S. baicalensis* | *S. baicalensis* |
| 0477006 | *S. baicalensis* | *S. baicalensis* |
| 0477012 | *S. baicalensis* | *S. baicalensis* |
| 0477026 | *S. baicalensis* | *S. baicalensis* |
| 0477027 | *S. baicalensis* | *S. baicalensis* |
| 0477058 | *S. baicalensis* | *S. baicalensis* |
| 002184 | ***S. baicalensis × S. krylovii*** | ***S. baicalensis*** |
| 0454628 | *S. baicalensis × S. krylovii* | *S. baicalensis × S. krylovii* |
| 0454720 | *S. baicalensis × S. krylovii* | *S. baicalensis × S. krylovii* |
| 002220 | *S. baicalensis × S. krylovii* | *S. baicalensis × S. krylovii* |
| 0451256 | ***S. capillata*** | ***S. capillata × S. krylovii*** |
| 0454736 | ***S. capillata*** | ***S. capillata × S. krylovii*** |
| 001544 | ***S. capillata*** | ***S. capillata × S. krylovii*** |
| 001394 | ***S. capillata*** | ***S. capillata × S. krylovii*** |
| 001079 | ***S. capillata*** | ***S. capillata × S. baicalensis*** |
| 001306 | ***S. capillata × S. krylovii*** | ***S. capillata*** |
| 001099 | ***S. capillata × S. krylovii*** | ***S. capillata*** |
| 001100 | ***S. capillata × S. krylovii*** | ***S. capillata*** |
| 001102 | ***S. capillata × S. krylovii*** | ***S. capillata*** |
| 001106 | ***S. capillata × S. krylovii*** | ***S. capillata*** |
| 001107 | ***S. capillata × S. krylovii*** | ***S. capillata*** |
| 001111 | ***S. capillata × S. krylovii*** | ***S. capillata*** |
| 001112 | ***S. capillata × S. krylovii*** | ***S. capillata*** |
| 000332 | ***S. capillata× S. krylovii*** | ***S. capillata*** |
| 000329 | ***S. capillata × S. baicalensis*** | ***S. capillata*** |
| 001399 | ***S. capillata × S. grandis*** | ***S. capillata × S. baicalensis*** |
| 002316 | *S. capillata* | *S. capillata* |
| 001310 | *S. capillata* | *S. capillata* |
| 001393 | *S. capillata* | *S. capillata* |
| 001395 | *S. capillata* | *S. capillata* |
| 0454271 | *S. capillata* | *S. capillata* |
| 0456693 | *S. capillata* | *S. capillata* |
| 0475125 | *S. capillata* | *S. capillata* |
| 0496240 | *S. capillata* | *S. capillata* |
| 001093 | *S. capillata* | *S. capillata* |
| 001094 | *S. capillata* | *S. capillata* |
| 001095 | *S. capillata* | *S. capillata* |
| 001097 | *S. capillata* | *S. capillata* |
| 001098 | *S. capillata* | *S. capillata* |
| 001108 | *S. capillata* | *S. capillata* |
| 001110 | *S. capillata* | *S. capillata* |
| 001117 | *S. capillata* | *S. capillata* |
| 001068 | *S. capillata* | *S. capillata* |
| 001071 | *S. capillata* | *S. capillata* |
| 001072 | *S. capillata* | *S. capillata* |
| 001074 | *S. capillata* | *S. capillata* |
| 001077 | *S. capillata* | *S. capillata* |
| 001078 | *S. capillata* | *S. capillata* |
| 001080 | *S. capillata* | *S. capillata* |
| 0451257 | *S. capillata* | *S. capillata* |
| 0454728 | *S. capillata* | *S. capillata* |
| 0454740 | *S. capillata* | *S. capillata* |
| 0454747 | *S. capillata* | *S. capillata* |
| 002505 | *S. capillata* | *S. capillata* |
| 000531 | *S. capillata* | *S. capillata* |
| 000539 | *S. capillata* | *S. capillata* |
| 000543 | *S. capillata* | *S. capillata* |
| 000547 | *S. capillata* | *S. capillata* |
| 000549 | *S. capillata* | *S. capillata* |
| 000555 | *S. capillata* | *S. capillata* |
| 000330 | *S. capillata* | *S. capillata* |
| 000331 | *S. capillata* | *S. capillata* |
| 000342 | *S. capillata* | *S. capillata* |
| 000343 | *S. capillata* | *S. capillata* |
| 000344 | *S. capillata* | *S. capillata* |
| 000827 | *S. capillata* | *S. capillata* |
| 000831 | *S. capillata* | *S. capillata* |
| 000838 | *S. capillata* | *S. capillata* |
| 000841 | *S. capillata* | *S. capillata* |
| 000842 | *S. capillata* | *S. capillata* |
| 000843 | *S. capillata* | *S. capillata* |
| 000849 | *S. capillata* | *S. capillata* |
| 002223 | *S. capillata* | *S. capillata* |
| 0457716 A | *S. glareosa* | *S. glareosa* |
| 0457716 B | *S. glareosa* | *S. glareosa* |
| 0454146 | *S. glareosa* | *S. glareosa* |
| 0454147 | *S. glareosa* | *S. glareosa* |
| 0426149 3 | *S. glareosa* | *S. glareosa* |
| 0426149 4 | *S. glareosa* | *S. glareosa* |
| 0426149 5 | *S. glareosa* | *S. glareosa* |
| 0426150 | *S. glareosa* | *S. glareosa* |
| 0477208 1 | *S. glareosa* | *S. glareosa* |
| 0477208 2 | *S. glareosa* | *S. glareosa* |
| 0477209 3 | *S. glareosa* | *S. glareosa* |
| 0477209 4 | *S. glareosa* | *S. glareosa* |
| 0477252 | *S. glareosa* | *S. glareosa* |
| 000438 | ***S. grandis*** | ***S. grandis × S. baicalensis*** |
| 000948 | ***S. grandis*** | ***S. grandis × S. krylovii*** |
| 000956 | ***S. grandis*** | ***S. grandis × S. krylovii*** |
| 000440 | ***S. grandis × S. krylovii*** | ***S. baicalensis*** |
| 000441 | ***S. grandis × S. krylovii*** | ***S. baicalensis*** |
| 000430 | *S. grandis* | *S. grandis* |
| 000432 | *S. grandis* | *S. grandis* |
| 000439 | *S. grandis* | *S. grandis* |
| 000444 | *S. grandis* | *S. grandis* |
| 000445 | *S. grandis* | *S. grandis* |
| 000460 | *S. grandis* | *S. grandis* |
| 000467 | *S. grandis* | *S. grandis* |
| 0454710 | *S. grandis* | *S. grandis* |
| 0454712 | *S. grandis* | *S. grandis* |
| 0454751 | *S. grandis* | *S. grandis* |
| 0454802 | *S. grandis* | *S. grandis* |
| 0454803 | *S. grandis* | *S. grandis* |
| 0454815 | *S. grandis* | *S. grandis* |
| 001396 | *S. grandis* | *S. grandis* |
| 001397 | *S. grandis* | *S. grandis* |
| 001398 | *S. grandis* | *S. grandis* |
| 000402 | *S. grandis* | *S. grandis* |
| 000405 | *S. grandis* | *S. grandis* |
| 000420 | *S. grandis* | *S. grandis* |
| 000421 | *S. grandis* | *S. grandis* |
| 000426 | *S. grandis* | *S. grandis* |
| 000427 | *S. grandis* | *S. grandis* |
| 000428 | *S. grandis* | *S. grandis* |
| 000360 | *S. grandis* | *S. grandis* |
| 000361 | *S. grandis* | *S. grandis* |
| 000363 | *S. grandis* | *S. grandis* |
| 000364 | *S. grandis* | *S. grandis* |
| 000365 | *S. grandis* | *S. grandis* |
| 000367 | *S. grandis* | *S. grandis* |
| 000368 | *S. grandis* | *S. grandis* |
| 0426162 | *S. grandis* | *S. grandis* |
| 0477312 | *S. grandis* | *S. grandis* |
| 0477313 | *S. grandis* | *S. grandis* |
| 0477314 | *S. grandis* | *S. grandis* |
| 0477315 | *S. grandis* | *S. grandis* |
| 0477316 | *S. grandis* | *S. grandis* |
| 0454748 | *S. grandis* | *S. grandis* |
| 0454754 | *S. grandis* | *S. grandis* |
| 001419 | *S. grandis* | *S. grandis* |
| 001420 | *S. grandis* | *S. grandis* |
| 002221 | *S. grandis* | *S. grandis* |
| 0477268 | *S. grandis* | *S. grandis* |
| 0477271 | *S. grandis* | *S. grandis* |
| 0477273 | *S. grandis* | *S. grandis* |
| 0477274 | *S. grandis* | *S. grandis* |
| 0477281 | *S. grandis* | *S. grandis* |
| 000944 | *S. grandis* | *S. grandis* |
| 000946 | *S. grandis* | *S. grandis* |
| 000949 | *S. grandis* | *S. grandis* |
| 000959 | *S. grandis* | *S. grandis* |
| 000963 | *S. grandis* | *S. grandis* |
| 000966 | *S. grandis* | *S. grandis* |
| 000967 | *S. grandis* | *S. grandis* |
| 0453556 | *S. grandis* | *S. grandis* |
| 0477278 | *S. grandis × S. krylovii* | *S. grandis × S. krylovii* |
| 0477279 | *S. grandis × S. krylovii* | *S. grandis × S. krylovii* |
| 000442 | ***S. krylovii*** | ***S. baicalensis × S. krylovii*** |
| 000443 | ***S. krylovii*** | ***S. baicalensis × S. krylovii*** |
| 0477007 | ***S. krylovii*** | ***S. baicalensis × S. krylovii*** |
| 0477008 | ***S. krylovii*** | ***S. baicalensis × S. krylovii*** |
| 0477011 | ***S. krylovii*** | ***S. baicalensis × S. krylovii*** |
| 0477060 | ***S. krylovii*** | ***S. baicalensis × S. krylovii*** |
| 000950 | ***S. krylovii*** | ***S. grandis × S. krylovii*** |
| 000449 | ***S. krylovii*** | ***S. grandis × S. krylovii*** |
| 001065 | ***S. krylovii*** | ***S. capillata × S. baicalensis*** |
| 001070 | ***S. krylovii*** | ***S. capillata × S. baicalensis*** |
| 001308 | ***S. krylovii × S. baicalensis*** | ***S. baicalensis*** |
| 001324 | ***S. krylovii × S. baicalensis*** | ***S. baicalensis*** |
| 0477832 | *S. krylovii* | *S. krylovii* |
| 000433 | *S. krylovii* | *S. krylovii* |
| 000435 | *S. krylovii* | *S. krylovii* |
| 000459 | *S. krylovii* | *S. krylovii* |
| 000461 | *S. krylovii* | *S. krylovii* |
| 000463 | *S. krylovii* | *S. krylovii* |
| 001392 | *S. krylovii* | *S. krylovii* |
| 001545 | *S. krylovii* | *S. krylovii* |
| 0451252 | *S. krylovii* | *S. krylovii* |
| 0451253 | *S. krylovii* | *S. krylovii* |
| 0454729 | *S. krylovii* | *S. krylovii* |
| 0454784 | *S. krylovii* | *S. krylovii* |
| 0454818 | *S. krylovii* | *S. krylovii* |
| 0478099 | *S. krylovii* | *S. krylovii* |
| 0478100 | *S. krylovii* | *S. krylovii* |
| 0478101 | *S. krylovii* | *S. krylovii* |
| 0478103 | *S. krylovii* | *S. krylovii* |
| 0477303 | *S. krylovii* | *S. krylovii* |
| 0477306 | *S. krylovii* | *S. krylovii* |
| 0477307 | *S. krylovii* | *S. krylovii* |
| 0477308 | *S. krylovii* | *S. krylovii* |
| 0477309 | *S. krylovii* | *S. krylovii* |
| 0477311 | *S. krylovii* | *S. krylovii* |
| 0454741 | *S. krylovii* | *S. krylovii* |
| 0454746 | *S. krylovii* | *S. krylovii* |
| 0454755 | *S. krylovii* | *S. krylovii* |
| 0454756 | *S. krylovii* | *S. krylovii* |
| 0454757 | *S. krylovii* | *S. krylovii* |
| 0454804 | *S. krylovii* | *S. krylovii* |
| 0454805 | *S. krylovii* | *S. krylovii* |
| 001421 | *S. krylovii* | *S. krylovii* |
| 0477265 | *S. krylovii* | *S. krylovii* |
| 0477269 | *S. krylovii* | *S. krylovii* |
| 0477270 | *S. krylovii* | *S. krylovii* |
| 0477275 | *S. krylovii* | *S. krylovii* |
| 0477280 | *S. krylovii* | *S. krylovii* |
| 0477282 | *S. krylovii* | *S. krylovii* |
| 0477285 | *S. krylovii* | *S. krylovii* |
| 000941 | *S. krylovii* | *S. krylovii* |
| 000942 | *S. krylovii* | *S. krylovii* |
| 000957 | *S. krylovii* | *S. krylovii* |
| 000969 | *S. krylovii* | *S. krylovii* |
| 000970 | *S. krylovii* | *S. krylovii* |
| 000972 | *S. krylovii* | *S. krylovii* |
| 0454646 | *S. krylovii* | *S. krylovii* |
| 0477207 | *S. krylovii* | *S. krylovii* |
| 000538 | *S. krylovii* | *S. krylovii* |
| 000582 | *S. krylovii* | *S. krylovii* |
| 000585 | *S. krylovii* | *S. krylovii* |
| 000586 | *S. krylovii* | *S. krylovii* |
| 000587 | *S. krylovii* | *S. krylovii* |
| 000588 | *S. krylovii* | *S. krylovii* |
| 000590 | *S. krylovii* | *S. krylovii* |
| 000591 | *S. krylovii* | *S. krylovii* |
| 000372 | *S. krylovii* | *S. krylovii* |
| 000373 | *S. krylovii* | *S. krylovii* |
| 000384 | *S. krylovii* | *S. krylovii* |
| 000385 | *S. krylovii* | *S. krylovii* |
| 000388 | *S. krylovii* | *S. krylovii* |
| 000391 | *S. krylovii* | *S. krylovii* |
| 000392 | *S. krylovii* | *S. krylovii* |
| 0495094 | *S. krylovii* | *S. krylovii* |
| 0495095 | *S. krylovii* | *S. krylovii* |
| 0495096 | *S. krylovii* | *S. krylovii* |
| 0495097 | *S. krylovii* | *S. krylovii* |
| 0495098 | *S. krylovii* | *S. krylovii* |
| 0495099 | *S. krylovii* | *S. krylovii* |
| 0495100 | *S. krylovii* | *S. krylovii* |
| 0468522 | *S. krylovii* | *S. krylovii* |
| 0495122 | *S. krylovii* | *S. krylovii* |
| 0470570 | *S. krylovii* | *S. krylovii* |
| 0470573 | *S. krylovii* | *S. krylovii* |
| 0469167 | *S. krylovii* | *S. krylovii* |
| 0469168 | *S. krylovii* | *S. krylovii* |
| 0496246 | *S. krylovii* | *S. krylovii* |
| 0469181 | *S. krylovii* | *S. krylovii* |
| 0469188 | *S. krylovii* | *S. krylovii* |
| 0469189 | *S. krylovii* | *S. krylovii* |
| 0469194 | *S. krylovii* | *S. krylovii* |
| 0469195 | *S. krylovii* | *S. krylovii* |
| 0469202 | *S. krylovii* | *S. krylovii* |

**Supplementary Figure S1**


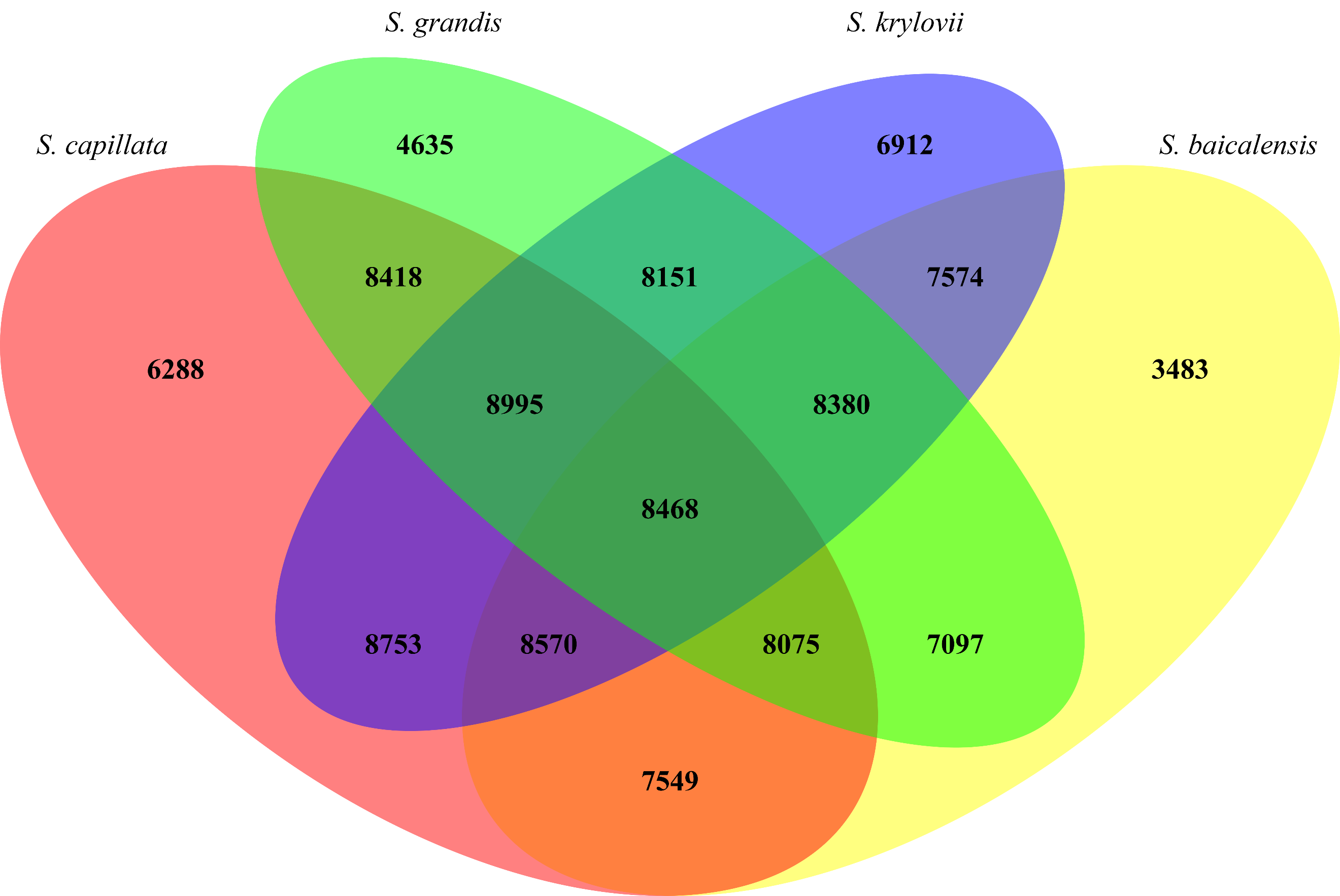


Venn diagram representing polymorphic SNPs among four pure *Stipa* species. The admixed individuals and *S. glareosa*, which did not show patterns of hybridisation, were omitted in the metric's calculation.

**Supplementary Figure S2**

**
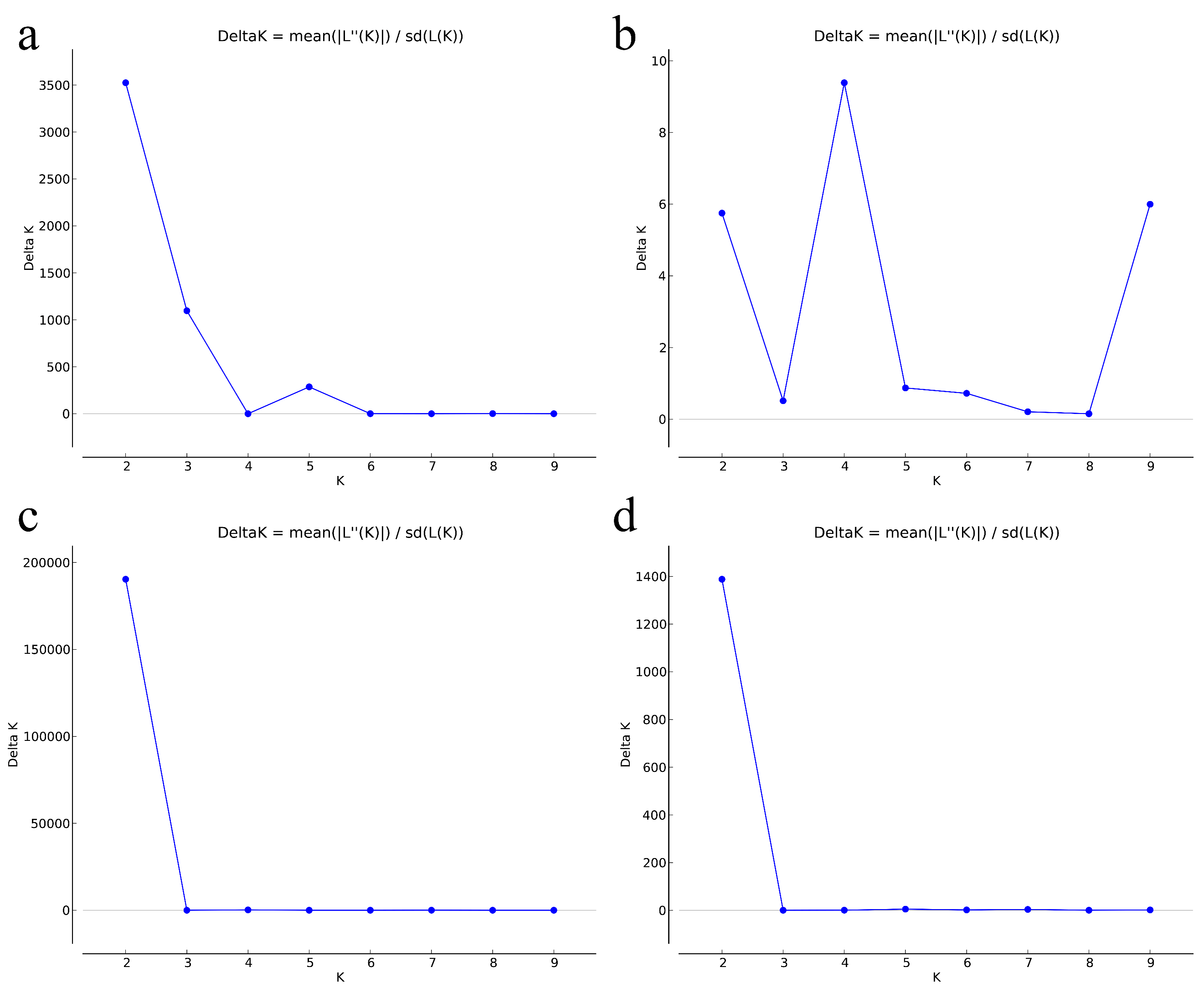
**

Delta K values calculated by Evanno's method across four species. (a) *S. baicalensis*. (b) *S. capillata.* (c) *S. grandis.* (d) *S. krylovii.*

**Supplementary Figure S3**


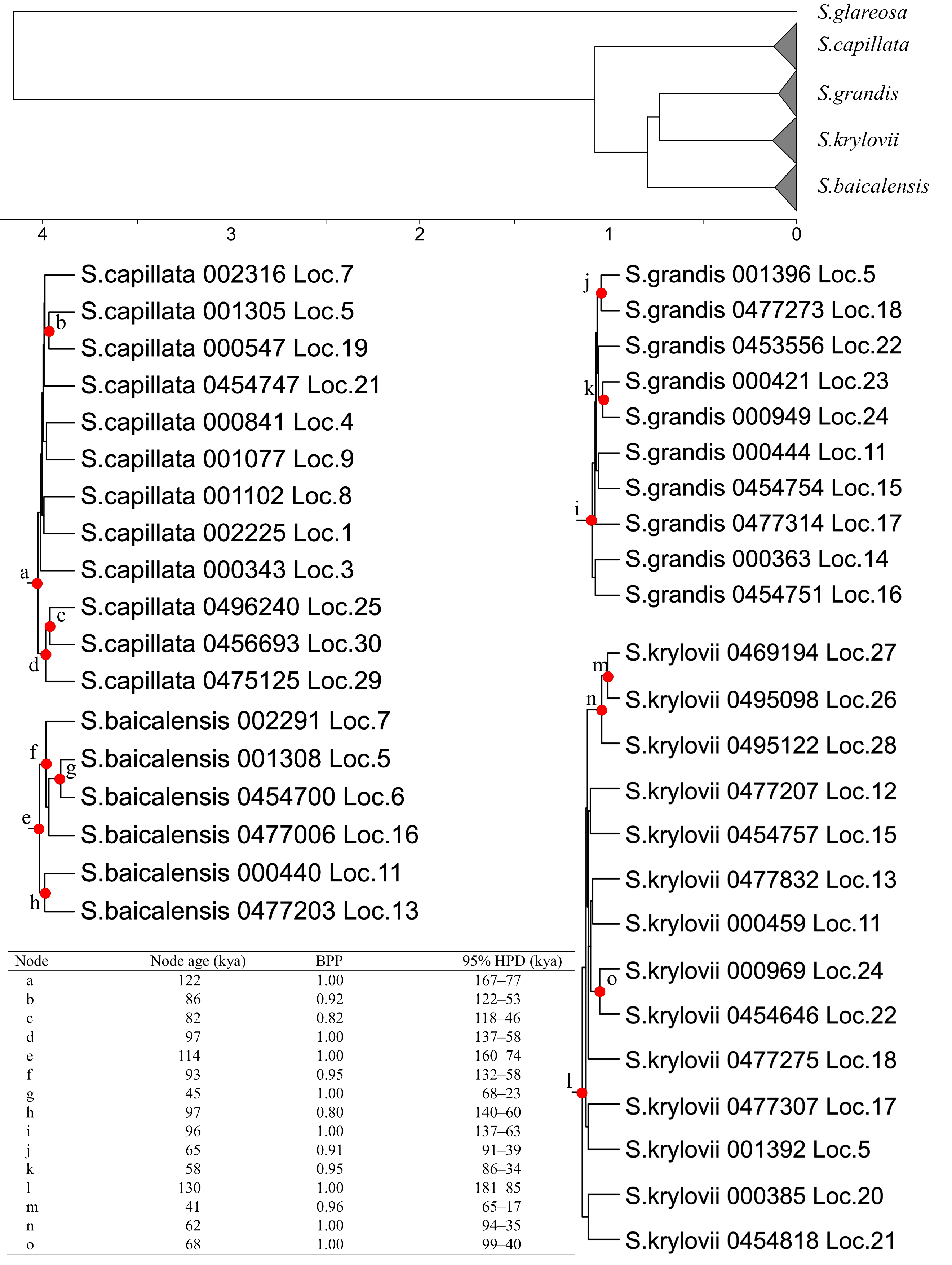


Phylogeny (at the top) and divergence date estimates at the species level (on the bottom) inferred by SNAPP. The scale shows divergence time in Mya. The red circles indicate nodes with the Bayesian posterior probabilities (BPP) ≥ 0.80. The lower-case letters refer to the embedded table containing data regarding the exact estimates of the divergence times (in kya), BPPs and 95% HPD intervals.

**Supplementary Figure S4**


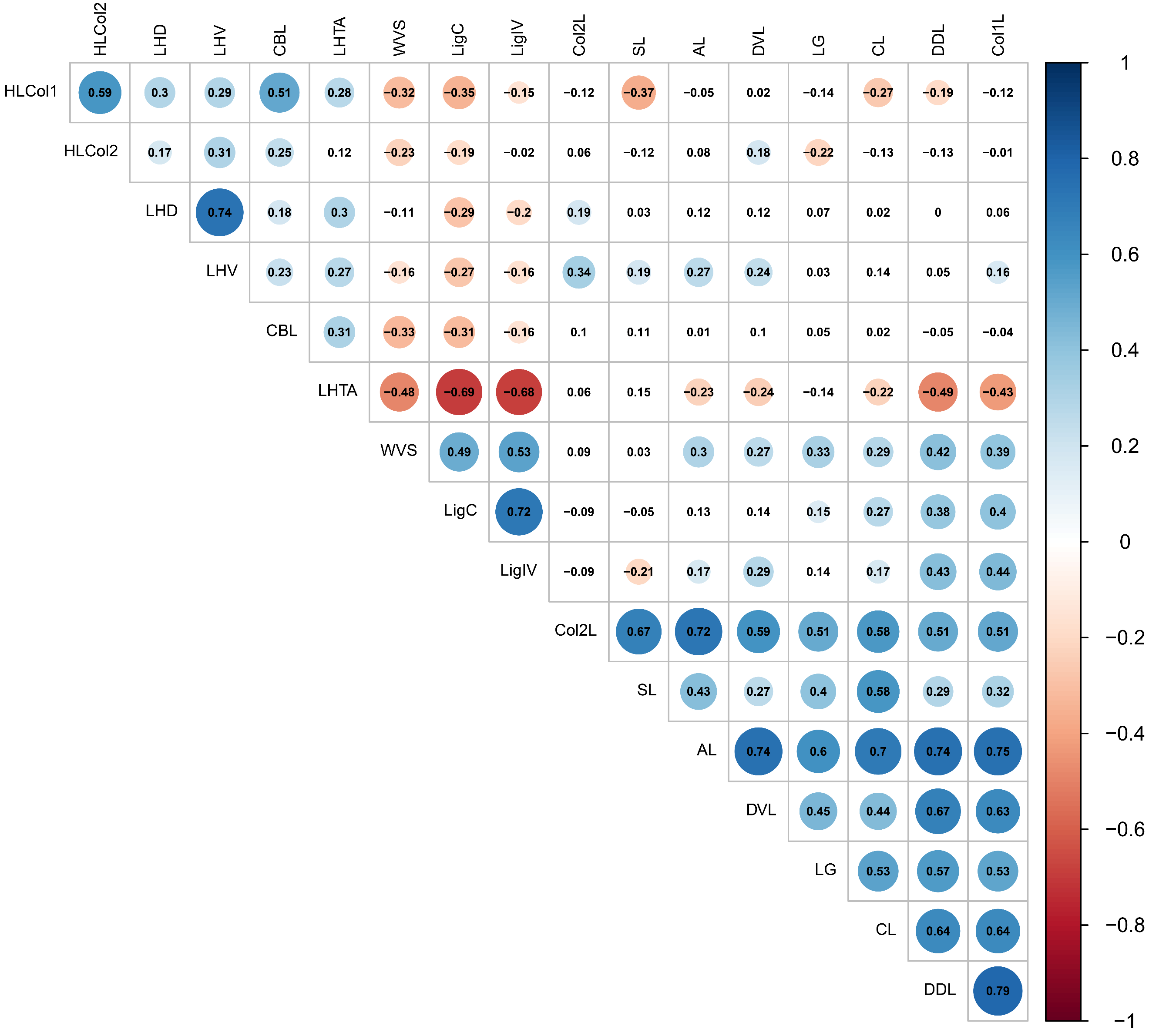


Correlation matrix of the studied morphological characters (abbreviations according to Table 3). Colour intensity and the size of the circle are proportional to the correlation coefficients (displayed in the circle). Positive correlations are blue while negative are red. All *p*-values of Pearson correlations were < 0.01.

**Supplementary Figure S5**

**
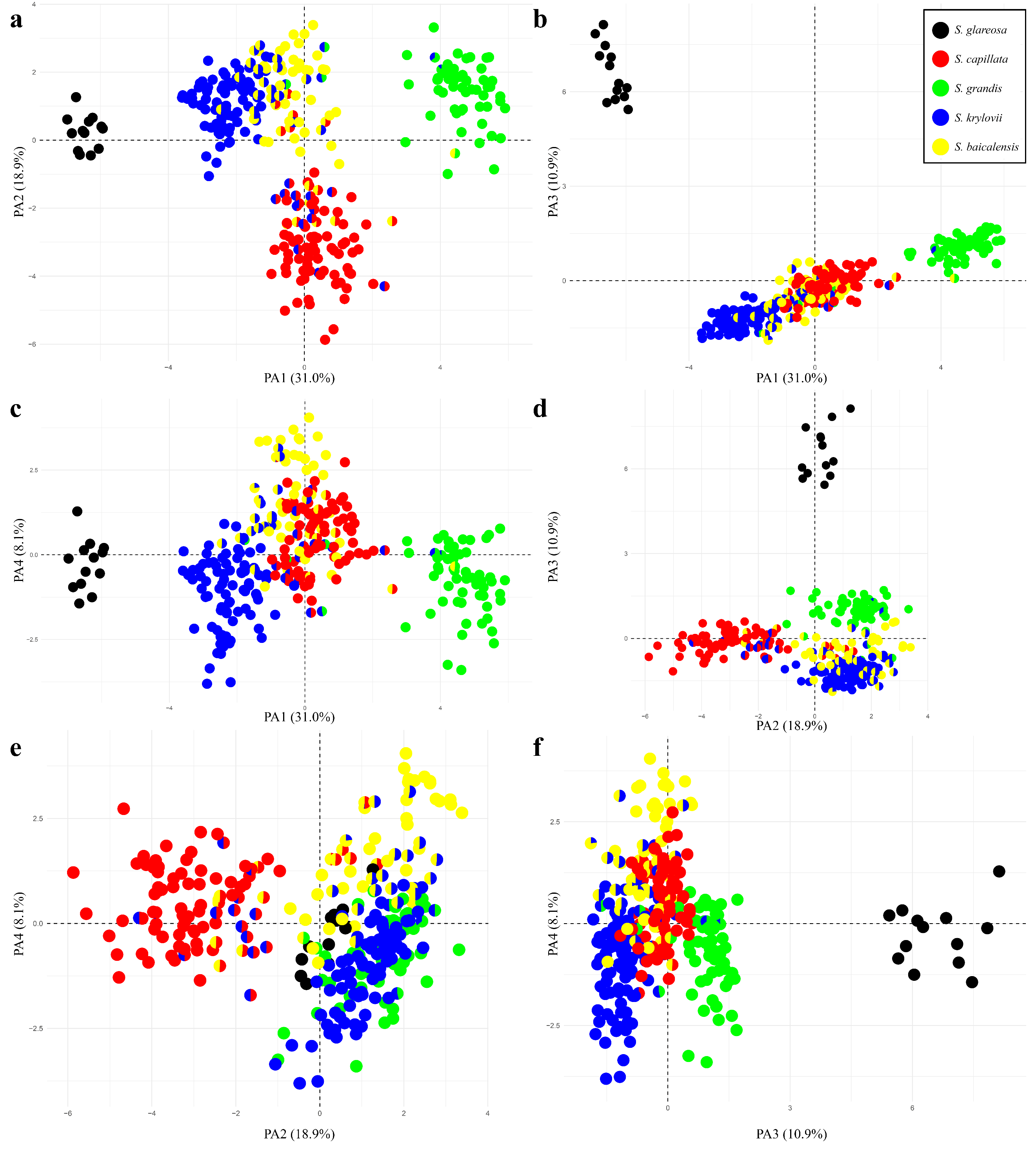
**

Factor analysis of mixed data performed on 17 quantitative and six qualitative characters of the five examined species of *Stipa*. (a) Plot of the principal axes one and two. (b) Plot of the principal axes one and three. (c) Plot of the principal axes one and four. (d) Plot of the principal axes two and three. (e) Plot of the principal axes two and four. (f) Plot of the principal axes three and four. The pie charts represent the proportions of membership established by fastSTRUCTURE for the best K=5.

**Supplementary Figure S6**

**
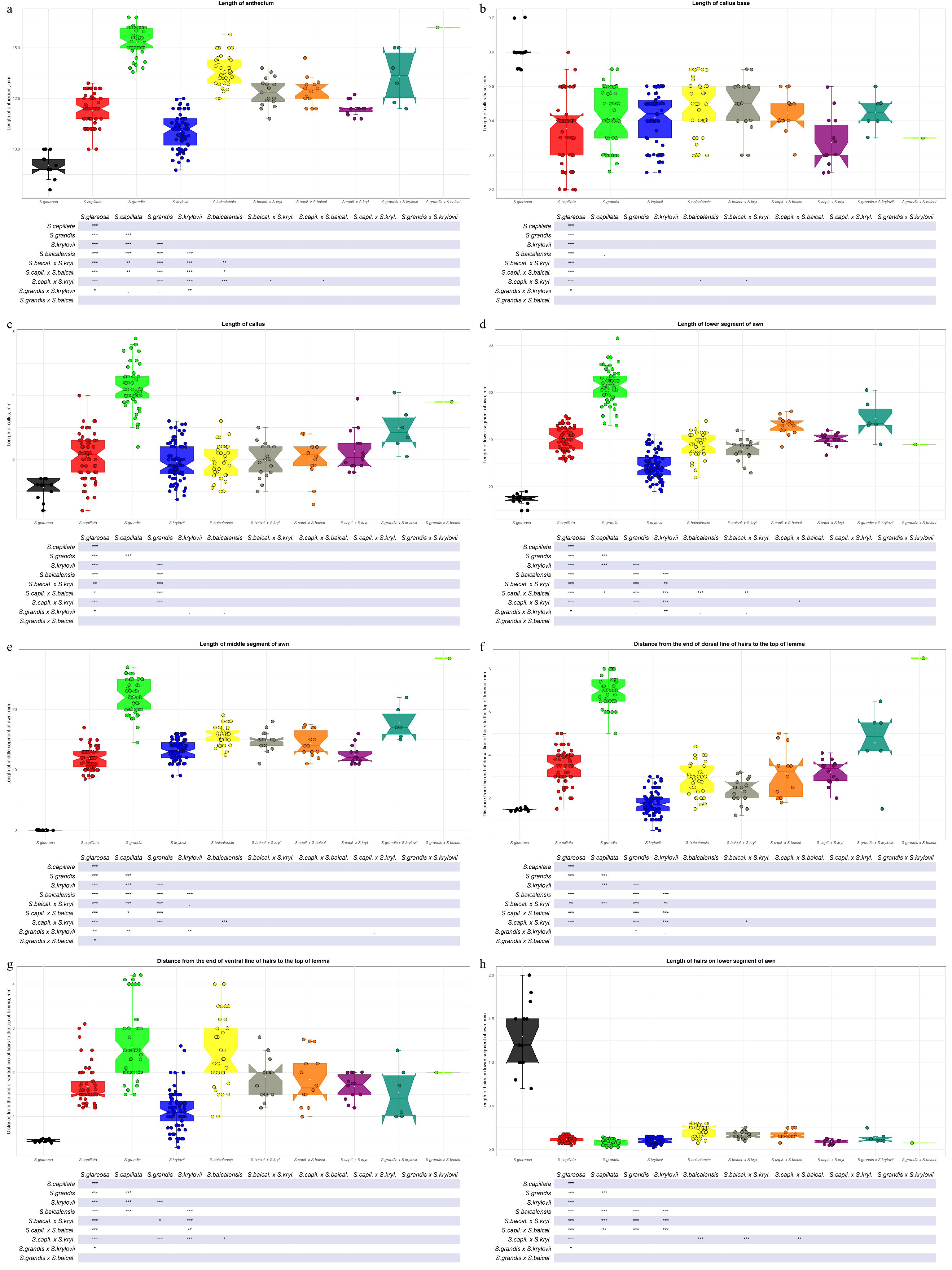
**


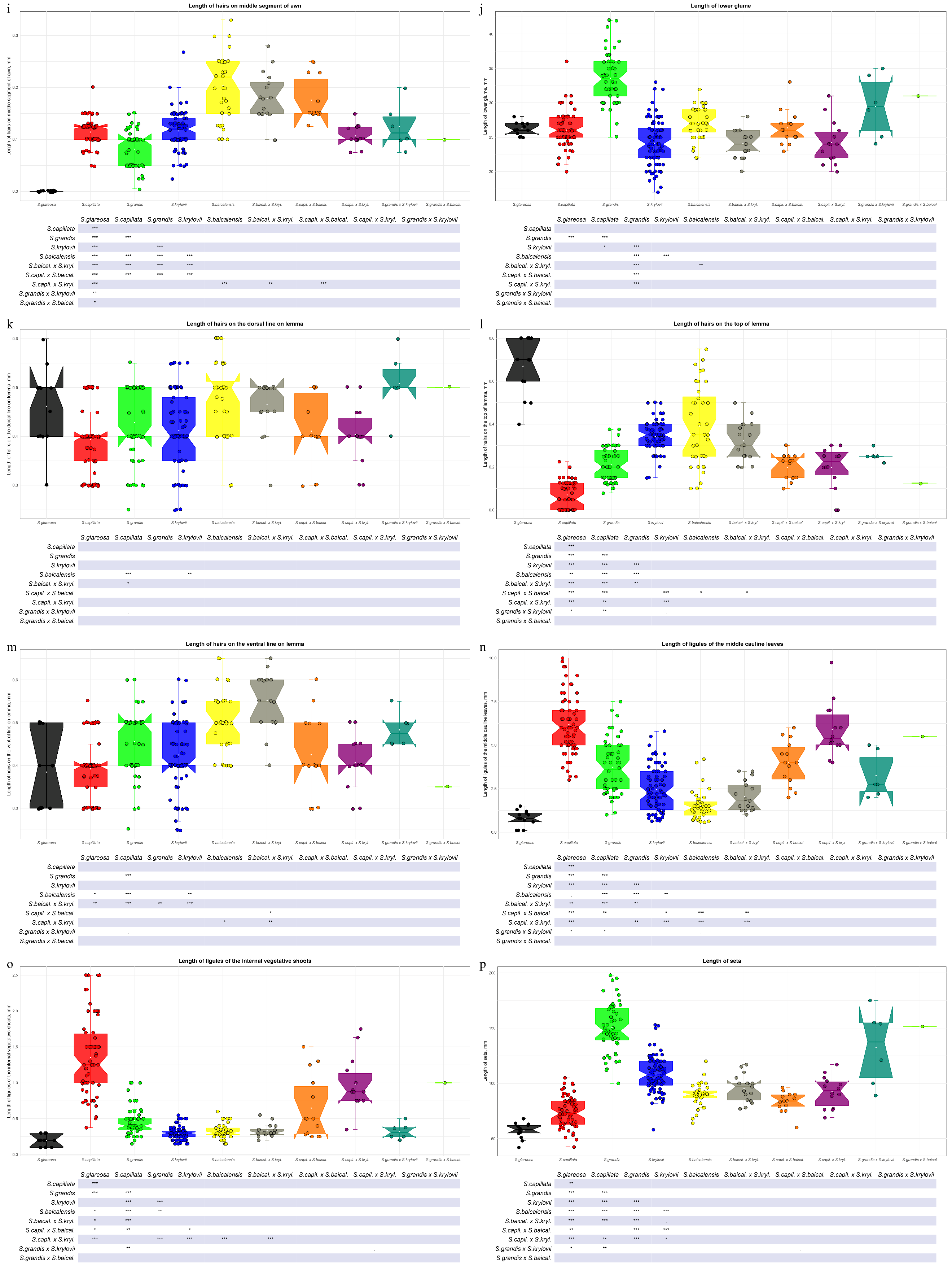


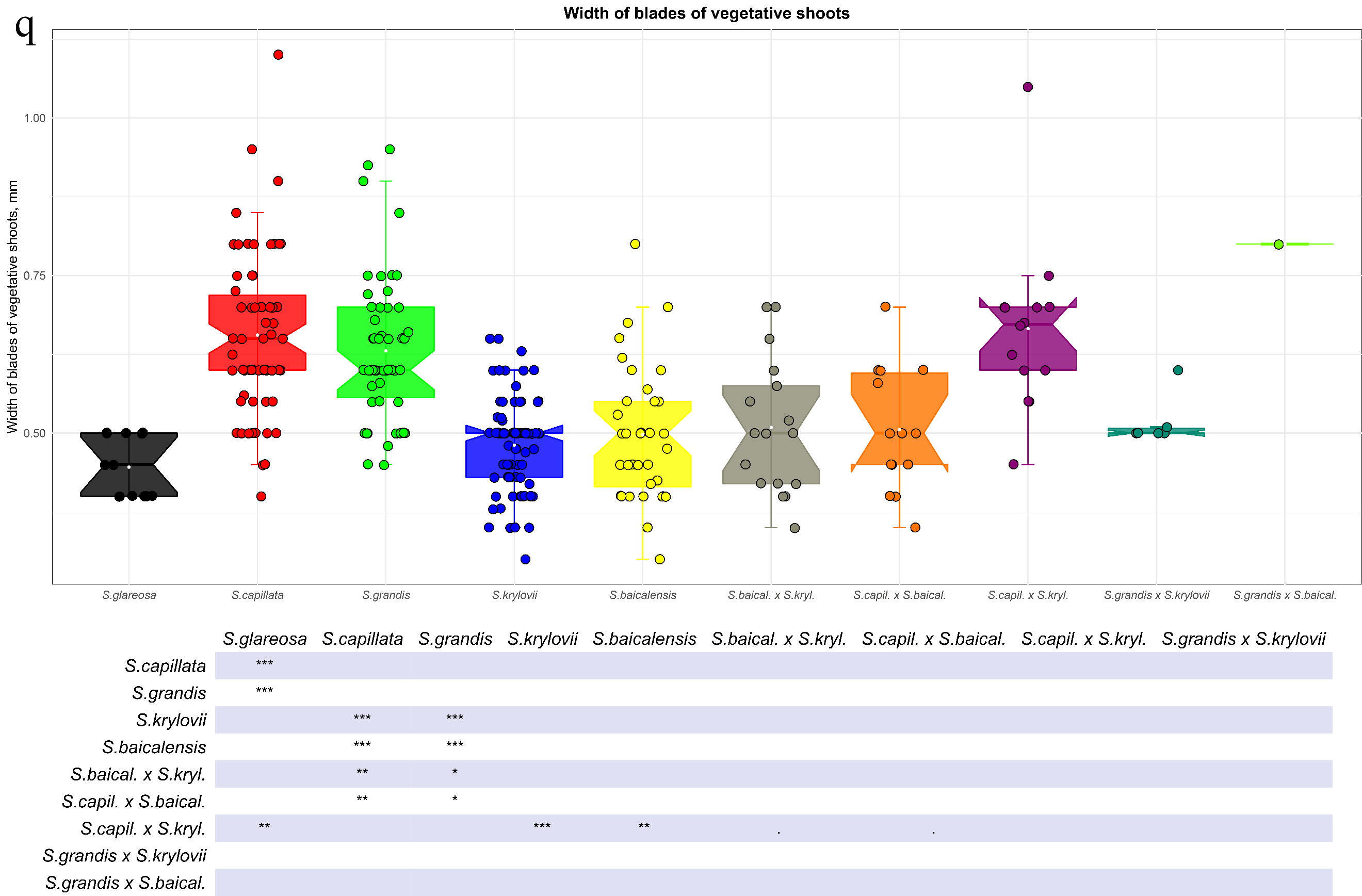


Notched boxplot demonstrating the mean (white circle), the median (dark black line), 95% confidence interval around the median (notch), inter-quartile ranges (25% to 75%), whiskers (5% and 95%) and minimum and maximum measurements (crosses) of quantitative characters (a-q) for the studied species. Statistical significance was tested by Wilcoxon rank-sum test for post hoc group comparisons with Bonferroni correction, *p* < 0.001, *p* < 0.01, *p* < 0.05, *p* < 0.1 and *p* < 1 noted as '***', '**', '*', '.' and no symbol, respectively. Due to the small sample size, *p*-values cannot be properly estimated for *S. grandis* × *S. krylovii* and *S. grandis* × *S. baicalensis*. Each dot represents an observation.

**Supplementary Figure S7**


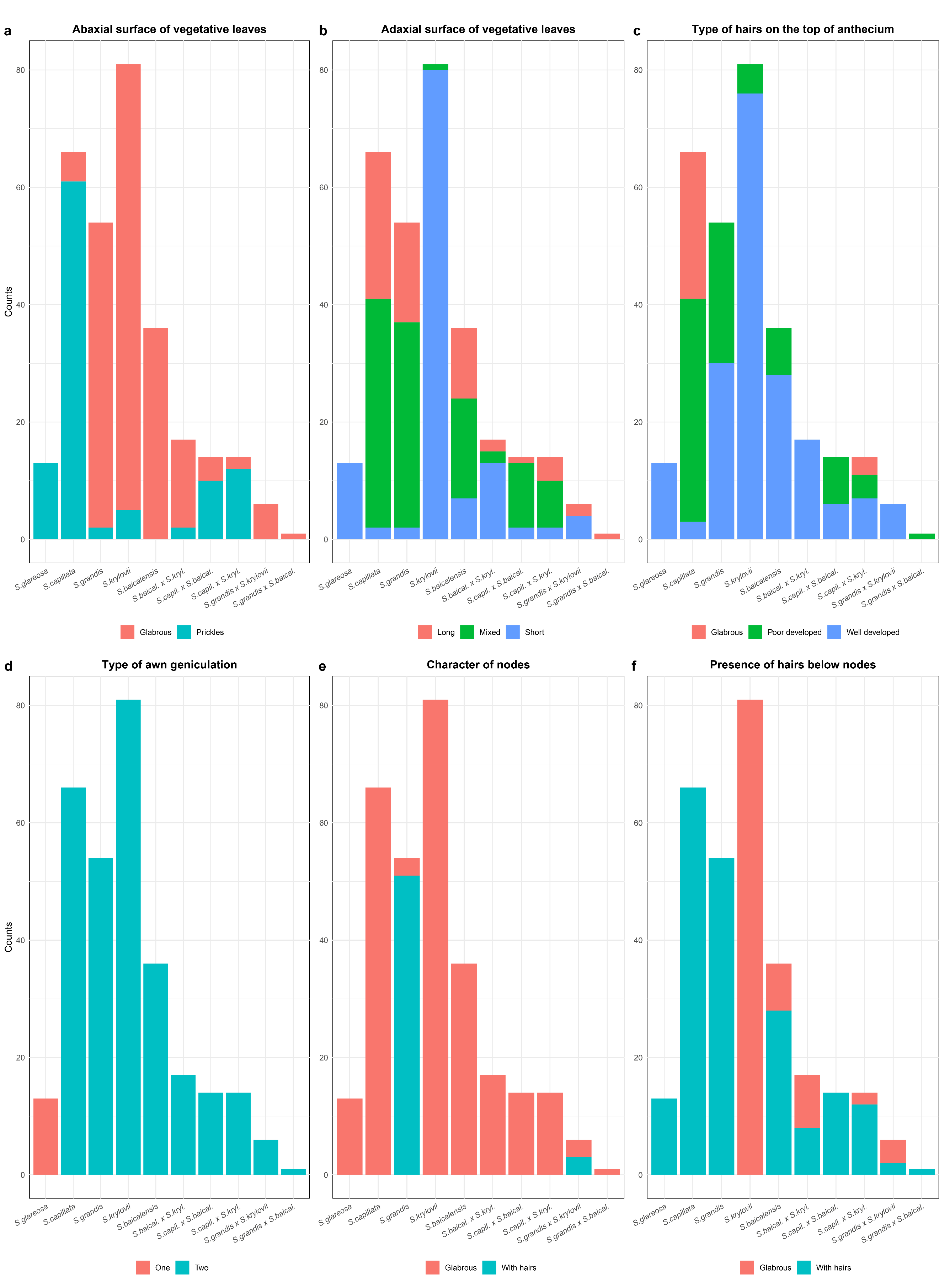


Bar charts displaying frequencies of the qualitative characters. (a) AbSVL. (b) AdSVL. (c) HTTA. (d) AG. (e) CN. (f) PHBN.
